# In-cell cryo-electron tomography reveals differential effects of type I and type II kinase inhibitors on LRRK2 filament formation and microtubule association

**DOI:** 10.7554/eLife.111075

**Published:** 2026-07-27

**Authors:** Tamar Basiashvili, Joshua Hutchings, Siyu Chen, Eva P Karasmanis, William Alexander Flaherty, Andres E Leschziner, Elizabeth Villa

**Affiliations:** 1 Department of Chemistry and Biochemistry, University of California San Diego La Jolla United States; 2 Aligning Science Across Parkinson's (ASAP) Collaborative Research Network Chevy Chase United States; 3 Howard Hughes Medical Institute Chevy Chase United States; 4 Department of Molecular Biology, University of California San Diego La Jolla United States; 5 Department of Cellular and Molecular Medicine, School of Medicine, University of California San Diego La Jolla United States; MRC Laboratory of Molecular Biology United Kingdom; Stanford University United States

**Keywords:** LRRK2, Parkinson's disease, cryo-ET, Human

## Abstract

Mutations in leucine-rich repeat kinase 2 (LRRK2) are a leading contributor to developing familial and idiopathic Parkinson’s disease (PD). Most PD-causing LRRK2 mutations increase the kinase activity, leading to increased phosphorylation of Rab GTPases, disrupting vesicular trafficking, cytoskeletal dynamics, and autophagy. Under homeostatic conditions, the bulk of WT and PD-mutant LRRK2 is found in the cellular cytosol. However, exogenously expressed LRRK2 can form microtubule-associated filaments that have been shown to affect molecular transport along microtubules in vitro. While the physiological relevance of microtubule binding has not been established yet, inhibitors being designed and tested as therapeutics have been shown to either promote or prevent filament formation of LRRK2. In this study, we examine the localization and resulting molecular organization of hyperactive LRRK2-I2020T, a common PD mutant, in HEK 293FT cells treated with type I (MLi-2) or type II (GZD-824) kinase inhibitors. Treatment with a type I kinase inhibitor results in extensive LRRK2-I2020T decoration around microtubules and microtubule bundling. Stabilization of LRRK2-I2020T filaments by type I inhibitor treatment allowed us to build a full-length closed-kinase model of LRRK2-I2020T in its cellular environment. Conversely, treatment with a type II inhibitor resulted in minimal microtubule decoration by LRRK2-I2020T compared to type I inhibitor-treated cells. This study provides a structural framework for understanding how type I and type II kinase inhibitors differentially modulate LRRK2 filament formation, demonstrating that type I inhibitor treatment promotes a distinct filament architecture, whereas such assemblies are not observed with type II inhibitors.

## Introduction

Parkinson’s disease (PD) is a chronic and progressive neurodegenerative disorder affecting approximately 10 million people worldwide (Kalia and Lang, 2015; Tolosa et al., 2020; Zimprich et al., 2004a; Ramesh and Arachchige, 2023). Genome-wide association studies have identified leucine-rich repeat kinase 2 (LRRK2) as a major risk factor for developing familial and idiopathic PD (Simón-Sánchez et al., 2009; Zimprich et al., 2004b; Funayama et al., 2002). LRRK2 contributes to disrupted cytoskeletal dynamics, lysosomal dysfunction, neuroinflammation, and impaired dopaminergic signaling (West et al., 2005; Manzoni et al., 2013; Alessi and Sammler, 2018; Steger et al., 2017).

**Figure 1.**
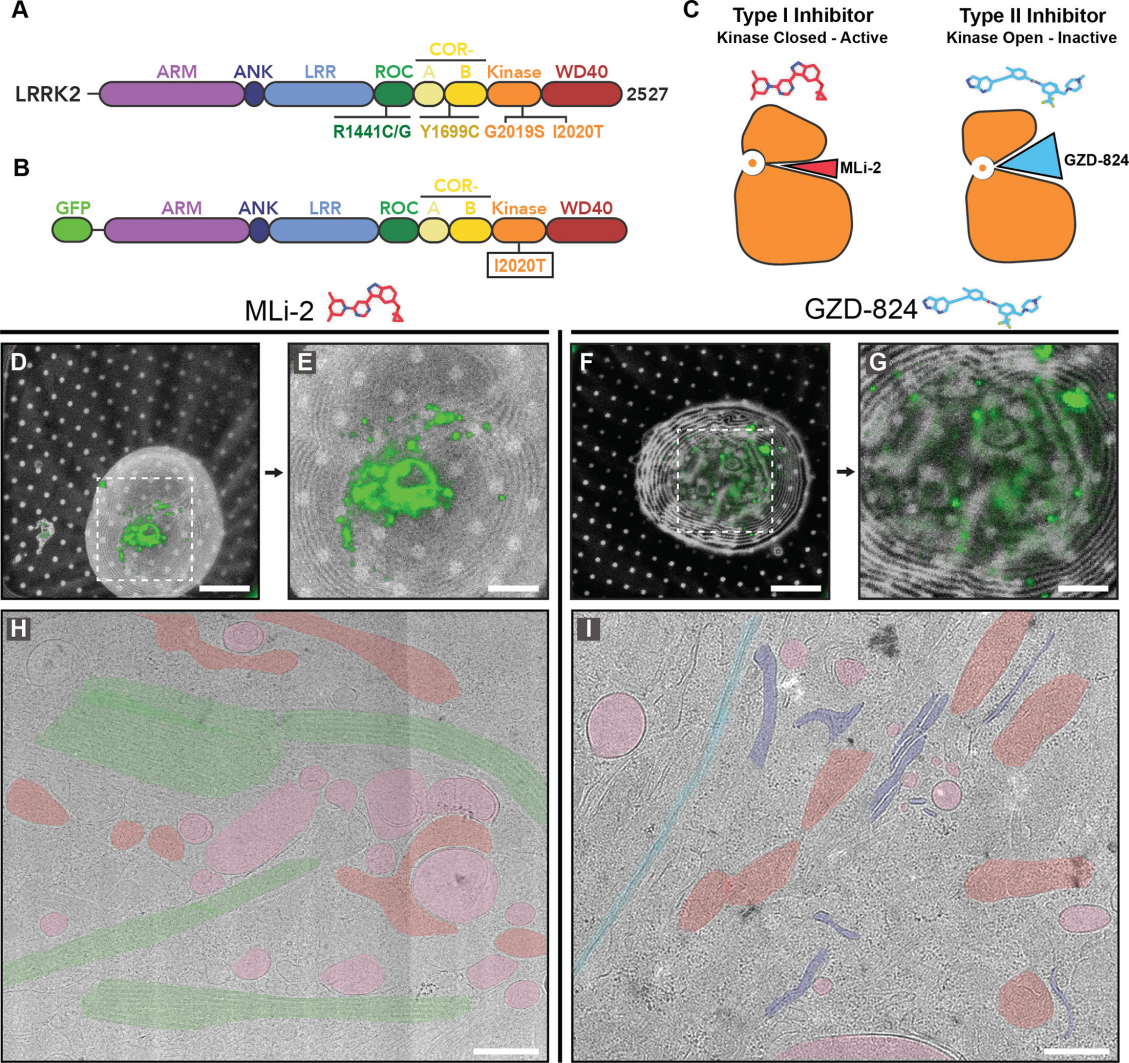
LRRK2^IT^ expression in 293T cells responds differently to treatment with type I and type II kinase inhibitors. (**A**) LRRK2 contains multiple functional domains, including armadillo (ARM), ankyrin repeats (ANK), leucine-rich repeats (LRR), Ras of complex (ROC), COR, kinase, and WD40 domain. (**B**) Schematic of the LRRK2 protein construct used in this paper, which includes a hyperactive I2020T mutation and an N-terminal GFP-tag. (**C**) Type I inhibitors (red) capture the LRRK2 kinase in active, closed conformation, while type II inhibitors (blue) trap the kinase of LRRK2 in an inactive, open-kinase conformation. (**D–E**) Cryo-fluorescence microscopy (cryo-FM) image of a vitreous cell on an electron microscopy grid expressing LRRK2^IT^ treated with MLi-2. The GFP-LRRK2^IT^ signal (green) is present as filaments as well as puncta. (**F–G**) Cryo-FM image of a cell expressing LRRK2^IT^ treated with GZD-824. The GFP-LRRK2^IT^ signal (green) shows a punctate distribution of the protein throughout the cytosol of the cell. (**H–I**) Cryo-TEM overview of the lamella of a cell expressing LRRK2^IT^ treated with MLi-2 (**H**) and GZD-824 (**I**). Microtubule bundles are highlighted in green, with some displaying dense LRRK2 decoration. Green: microtubule bundles; red: mitochondria; pink: vesicles; blue: plasma membrane; purple: endoplasmic reticulum. Scale bars: D, F, 20 μm; E, G, 5 μm; H, I, 500 nm.

LRRK2 is a large multidomain protein, one in four in the human genome to contain a GTPase and a kinase (Alessi and Pfeffer, 2024; Leschziner, 2025; Berwick et al., 2019). Its C-terminal catalytic region is composed of ROC GTPase, COR, kinase, and WD40 (RCKW) domains (Figure 1A). Pathogenic mutations in this region (e.g. I2020T, G2019S, R1441H/G/C/S, Y1699C) increase the kinase activity, leading to hyperphosphorylation of Rab GTPases disrupting fusion, docking, and trafficking of vesicular membranes (Bonet-Ponce and Cookson, 2022; Di Maio et al., 2018; Gloeckner et al., 2006; Steger et al., 2016). The N-terminal region mediates protein-protein interactions, facilitating recognition of LRRK2 substrates through the leucine-rich repeats (LRR), ankyrin (ANK), and armadillo (ARM) domains (Figure 1A). In the autoinhibited inactive kinase state, the N-terminus occludes catalytic domains and limits substrate access to the kinase domain (Sanz Murillo et al., 2023; Chen et al., 2025; Zhu et al., 2024). Upon activation, these N-terminal repeats are proposed to undock, enabling substrate binding near the kinase moiety and subsequent phosphorylation of the target (Sanz Murillo et al., 2023). Recent structural studies have elucidated the architecture of LRRK2’s catalytic RCKW domains in both active and inactive conformations, as well as full-length autoinhibited and intermediate activation state structures of the protein (Sanz Murillo et al., 2023; Chen et al., 2025; Zhu et al., 2024; Watanabe et al., 2020; Deniston et al., 2020; Snead et al., 2022; Myasnikov et al., 2021; Zhu et al., 2023; Martinez Fiesco et al., 2025). Resolving the full-length closed-kinase structure of LRRK2 remains a significant challenge due to the large conformational heterogeneity of the N-terminal repeats in the active state.

**Figure 1—figure supplement 1.**
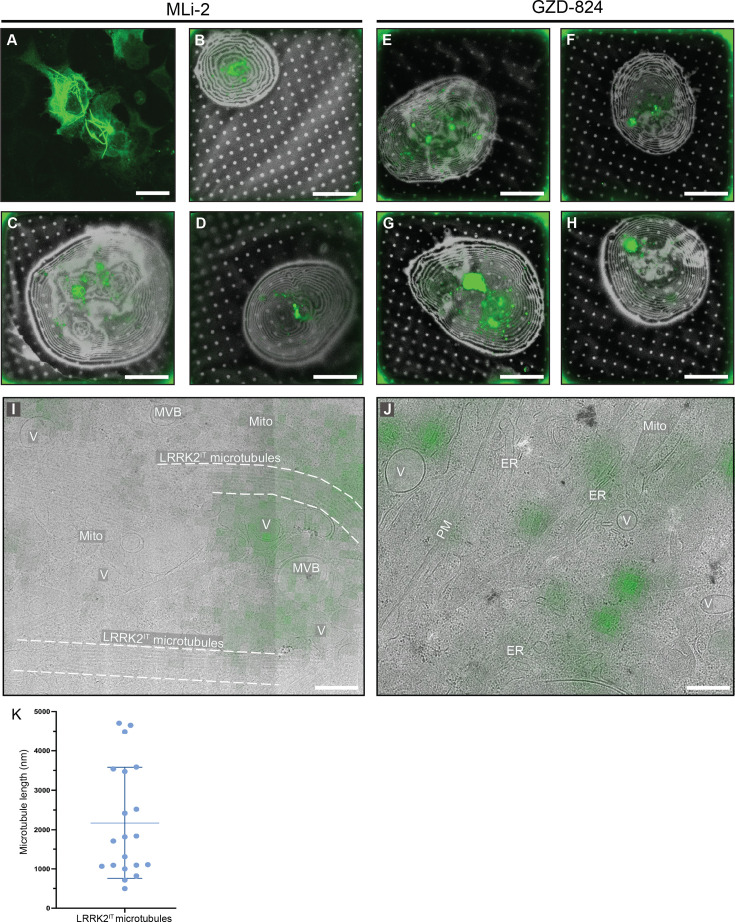
LRRK2^IT^ expression in 293T cells responds differently to treatment with type I and type II kinase inhibitors. (**A**) LRRK2^IT^ microtubule-associated filaments (green) in 293T fixed cells treated with MLi-2. (**B–D**) Cryo-fluorescence micrographs of GFP-LRRK2^IT^-expressing cells treated with MLi-2 show a distribution of LRRK2^IT^ as filaments, puncta, and diffuse in the cytosol. (**E–H**) Cryo-fluorescence microscope images of GFP-LRRK2^IT^-expressing cells treated with GZD-824 displaying diffuse and punctate distribution of LRRK2^IT^. (**I–J**) Cryo-TEM lamella overview of LRRK2^IT^-expressing cell treated with either MLi-2 (**I**) or GZD-824 (**J**). GFP-LRRK2^IT^ signal (green) is overlaid from cryo-FM imaging to guide localization of LRRK2^IT^. (**K**) Length of LRRK2^IT^ filaments evaluated from the cryo-TEM overview in (**I**) cells treated with MLi-2. Each data point represents a single filament. (22 data points; vertical bar represents mean with standard deviation 2.17±1.4 μm). MVB: multivesicular body V: vesicles, ER: endoplasmic reticulum, Mito: mitochondria, PM: plasma membrane. Scale bars: A, 40 μm; B–H, 20 μm; I–J, 500 nm.

Given its role in PD pathogenesis, LRRK2 has been the focus of small-molecule kinase inhibitor development, with two drug candidates currently in clinical testing (Tang et al., 2023; Raig et al., 2025; Hu et al., 2023; Ren et al., 2013). There are two major classes of LRRK2 inhibitors: type I LRRK2-specific inhibitors (e.g. MLi2, LRRK2-IN-1, HG-10-102-01) stabilize the kinase domain in its closed, active-like conformation, while type II broad-spectrum inhibitors (e.g. GZD-824, ponatinib, rebastinib) stabilize the kinase in its open, inactive-like conformation (Ren et al., 2013; Deng et al., 2011; Tasegian et al., 2021; Scott et al., 2017; Choi et al., 2012; Fell et al., 2015; Estrada et al., 2014; Figure 1C). Both types of inhibitors bind to the kinase domain, but type I inhibitors compete for ATP binding, while type II inhibitors bind to the allosteric site adjacent to the kinase domain (Sanz Murillo et al., 2023; Zhu et al., 2024). The conformational changes of the kinase domain when treated with different types of inhibitors have been characterized, but the effect of inhibitor treatment on LRRK2 localization and molecular architecture in cells remains unclear (Sanz Murillo et al., 2023; Zhu et al., 2024).

In cells, LRRK2 is typically cytosolic, but under certain conditions, it binds to microtubules and forms extensive filaments (Watanabe et al., 2020; Kett et al., 2012; Schmidt et al., 2021). Association with microtubules disrupts molecular transport along microtubules in vitro (Deniston et al., 2020). Hyperactivating pathogenic mutations such as I2020T and R1441H/G/C/S increase the protein’s propensity to form microtubule-associated filaments (Watanabe et al., 2020; Deniston et al., 2020; Snead et al., 2022). Additionally, type I, but not type II, kinase inhibitor treatment increases LRRK2 filamentation in cells (Snead et al., 2022; Dederer et al., 2024). While the physiological significance of LRRK2 filamentation remains unclear, microtubule association provides an assay to test the effects of kinase inhibitors on the molecular architecture of LRRK2 in cellular models.

In this study, we investigate the effects of different types of kinase inhibitors on the cellular architecture and localization of PD-associated LRRK2-I2020T (LRRK2^IT^). To this end, we expressed GFP-tagged LRRK2^IT^ in 293T cells and treated samples with either type I (MLi-2) or type II (GZD-824) inhibitors. To visualize the effects of inhibitor treatment on LRRK2 architecture at nanometer resolution, we used cryo-correlative light and electron microscopy (cryo-CLEM), which combines cryo-electron tomography (cryo-ET) with cryo-fluorescence microscopy (cryo-FM) (Lam and Villa, 2021; Pyle and Zanetti, 2021; Villa et al., 2013).

In cells treated with MLi-2, we observed highly ordered LRRK2-decorated microtubules that formed extensive microtubule bundles of 15–30 microtubules. In contrast, LRRK2 microtubule association was rarely observed in GZD-824-treated cells. These observations point to distinct mechanisms by which type I and type II kinase inhibitors influence LRRK2 filamentation in a cellular context. Besides increased filament formation, MLi-2 treatment reduced conformational heterogeneity of LRRK2^IT^, allowing us to characterize the architecture of the full-length microtubule-associated conformation of LRRK2^IT^. We present the structure of full-length LRRK2^IT^ in a closed-kinase conformation and N-terminal repeats undocked from the RCKW domains, providing insight about hyperactive protein architecture in the cellular environment.

## Results

### LRRK2^IT^ localizes to microtubules in the presence of a type I inhibitor and to the cytosol with a type II inhibitor

We used cryo-CLEM to study how cellular localization of GFP-tagged hyperactive LRRK2^IT^ is affected by treatment with either type I (MLi-2) or type II (GZD-824) inhibitors (Figure 1; Figure 1—figure supplement 1). Our previous work showed that expression of LRRK2^IT^ in 293T cells forms an extensive network of filaments around microtubules (Watanabe et al., 2020). Here, we added either MLi-2 or GZD-824 inhibitors that resulted in significant phenotypic differences in LRRK2^IT^ localization (Figure 1D–G; Figure 1—figure supplement 1A–J). In cells treated with MLi-2, we observed LRRK2^IT^ in extended filaments, puncta, and diffuse in the cytosol (Figure 1D–E; Figure 1—figure supplement 1A–D). In contrast, when cells were treated with GZD-824, LRRK2^IT^ was mostly localized to puncta and distributed throughout the cytosol, with reduced filament formation (Figure 1F–G; Figure 1—figure supplement 1E–H), in agreement with our previous work (Deniston et al., 2020; Snead et al., 2022; Dederer et al., 2024).

To probe this difference at the structural level, we used cryo-focused ion beam (cryo-FIB) milling to produce thin samples of vitrified cells for cryo-ET. Cryo-FM images were aligned to cryo-TEM overviews of the lamella to localize LRRK2^IT^ (Figure 1H–I; Figure 1—figure supplement 1I–J). In cells treated with MLi-2, we co-localized LRRK2^IT^ with an extended network of microtubules (Figure 1H–I; Figure 1—figure supplement 1I–J). These microtubules were coated with a dense LRRK2^IT^ lattice and bundled. The LRRK2^IT^-decorated microtubule network was extensive, spanning 2.17±1.40 μm in length (Figure 1H; Figure 1—figure supplement 1K). By contrast, microtubule bundling was not present in GZD-824-treated cells (Figure 1I).

### Type I kinase inhibitor treatment promotes bundling of LRRK2^IT^-decorated microtubules

**Figure 2.**
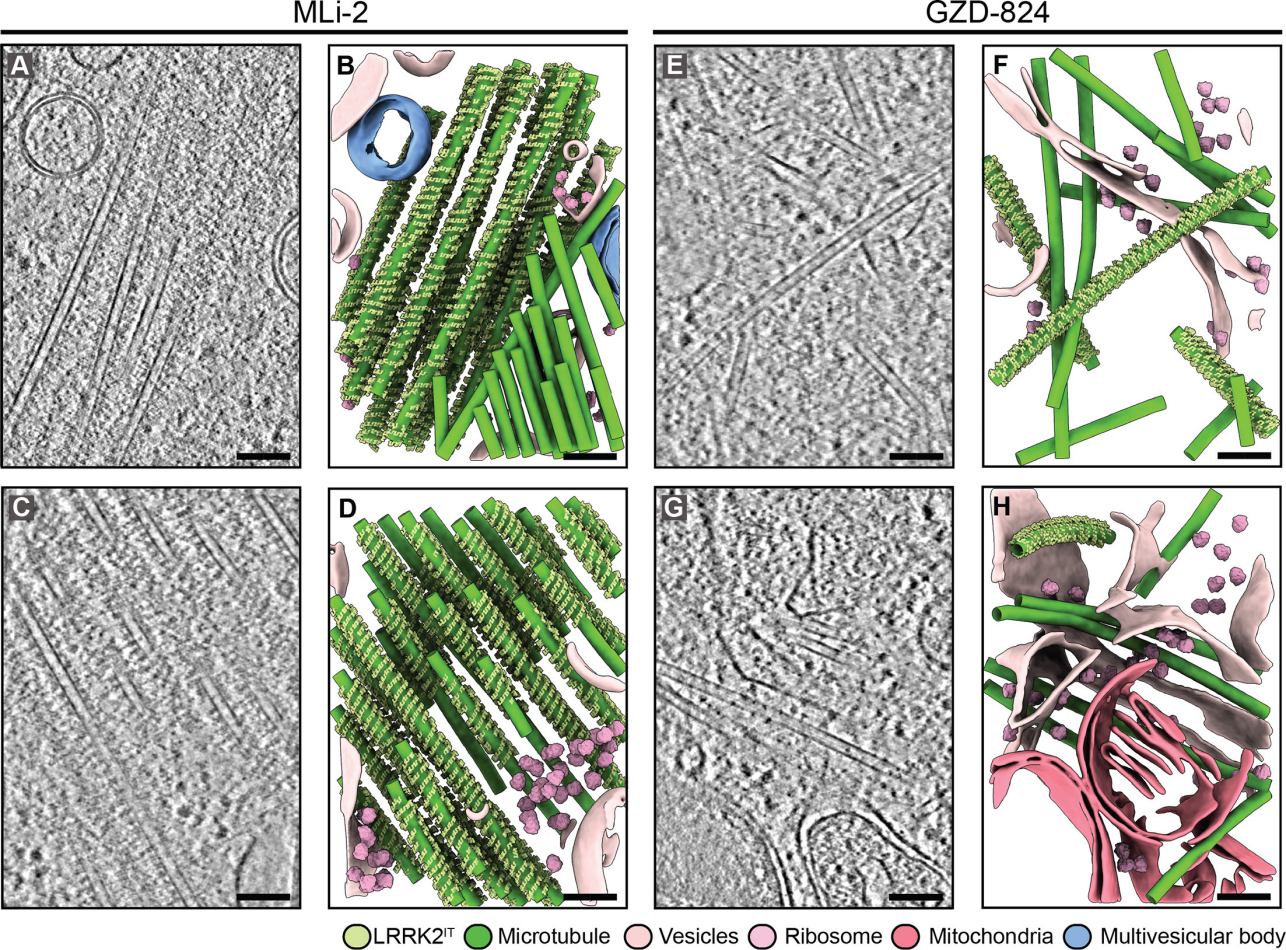
Treatment with the type I kinase inhibitor MLi-2 promotes the formation of higher-order LRRK2^IT^-decorated microtubule bundles, whereas the type II kinase inhibitor GZD-824 reduces LRRK2^IT^ filamentation and microtubule bundling in cells. (**A, C**) Representative tomogram slices through the cytosol of a cell treated with MLi-2, showing a bundle of microtubules decorated with LRRK2^IT^. (**B, D**) Segmentation of the tomogram in A, C, highlighting the bundles of microtubules decorated with a well-ordered LRRK2^IT^ lattice. (**E**) Tomogram slice of a cell treated with GZD-824, showing sparse LRRK2^IT^ decoration on microtubules. (**F**) Segmentation of the tomogram in E. LRRK2^IT^ decoration is present on three microtubules while there are nine undecorated microtubules present in the proximity. (**G**) Representative image of a cell treated with GZD-824. Single microtubule is decorated with LRRK2^IT^. This distribution pattern contrasts sharply with the extensive microtubule-associated filaments observed following type I inhibitor treatment. (**H**) Segmentation of a tomogram in G of a cell treated with GZD-824. Six undecorated microtubules are present near a single LRRK2-decorated microtubule. Lime green: LRRK2^IT^; green: microtubule; red: mitochondria; light pink: vesicles; blue: multivesicular bodies; pink: ribosomes. Scale bars: 100 nm.

**Figure 3.**
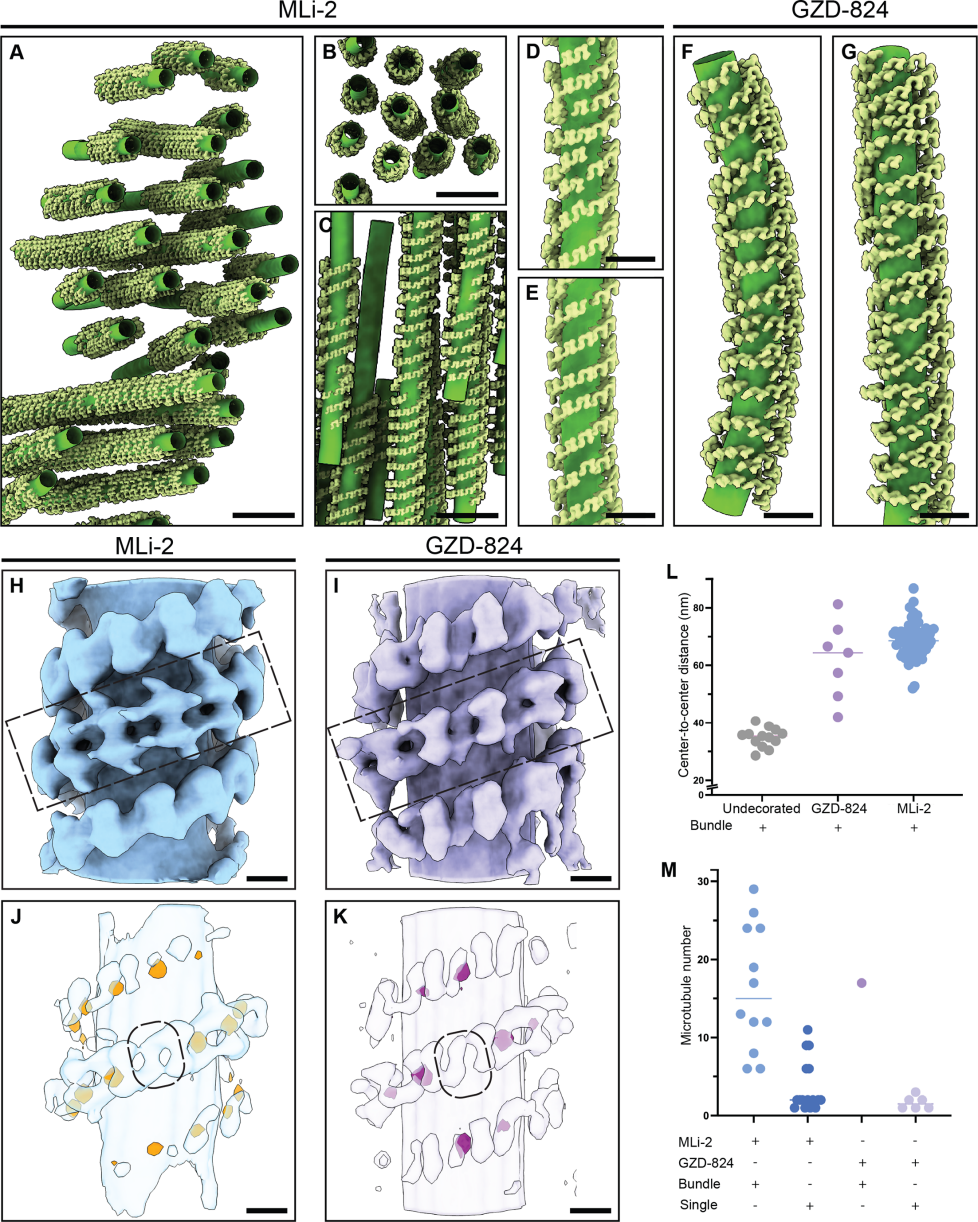
LRRK2^IT^ lattice that decorates microtubules is highly ordered in cells treated with type I as compared to type II inhibitor. (**A**) Segmented representation of an LRRK2^IT^-associated microtubule bundle in MLi-2-treated cells. Individual LRRK2^IT^ subunits are highly ordered around the microtubules. (**B**) Top-down view of the segmented representation of an LRRK2^IT^-associated microtubule bundle in MLi-2-treated cells. The individual microtubules are separated from each other by 65–70 nm. (**C**) Segmented representation of an LRRK2^IT^-associated microtubule bundle in MLi-2-treated cells. (**D–E**) Close-up view of the LRRK2^IT^ decoration on microtubules in MLi-2-treated cells. (**F–G**) Segmented representation of LRRK2^IT^-associated microtubules in GZD-824-treated cells. LRRK2^IT^ lattice is less ordered compared to MLi-2-treated lattice in (**D–E**). (**H–I**) Subtomogram average of the LRRK2^IT^ associated with microtubules from MLi-2 (**H**) or GZD-824 (**I**) treated cells. MLi-2-treated cells exhibit a more ordered lattice of LRRK2^IT^ than the lattice found in GZD-824-treated cells. (**J–K**) Nearest-neighbor analysis of a central LRRK2^IT^ subunit (highlighted in black) in MLi-2- and GZD-824-treated cells. In MLi-2-treated cells, the central LRRK2^IT^ subunit has 16 nearest neighbors highlighted in orange. While in GZD-824-treated cells, the central LRRK2^IT^ subunit has only eight nearest neighbors shown in pink. (**L**) Evaluation of the center-to-center distance of undecorated and LRRK2^IT^-decorated microtubule bundles in MLi-2- and GZD-824-treated cells (MLi-2 n=80, GZD-824 n=7, undecorated n=13; vertical line at median). (**M**) Quantification of LRRK2^IT^-decorated microtubule (MT) bundles and isolated microtubules in MLi-2- and GZD-824-treated cells (bundles quantified in MLi-2-treated cells n=12, isolated MTs quantified in MLi-2-treated cells n=16, isolated MTs quantified in GZD-824 n=6, bundle of MTs quantified in GZD-824 bundle n=1, vertical line at median). Scale bars: A–C, 40 nm; D–G, 20 nm; H–K, 10 nm.

We acquired cryo-electron tomograms of MLi-2- and GZD-824-treated cells targeting LRRK2^IT^ localization sites and identified all microtubules within the data. In MLi-2-treated cells, we focused on microtubules that were decorated with an LRRK2^IT^ lattice (Figure 2A–D; Figure 2—figure supplement 1A–B; Figure 2—figure supplement 2). We found a substantial fraction of them to be surrounded by other decorated microtubules, often closely spaced and arranged in parallel, suggesting that LRRK^IT^ binding promotes microtubule bundling (Figure 2A–D; Figure 2—figure supplement 1A–B). Bundles of LRRK2^IT^-decorated microtubules contained an average of 16±8.04 microtubules (range 5–30; Figure 3M). The center-to-center distance between LRRK2^IT^-decorated microtubules was 68.7 nm ±5.73 nm, while undecorated microtubule bundles had a center-to-center distance of 35 nm ±3.00 nm in our data and in previous findings (Figure 3L; Watanabe et al., 2020). The expanded spacing observed in the LRRK2^IT^-decorated microtubules is due to the 15-nm-thick LRRK2^IT^ layer that coats each microtubule, adding 30 nm to the center-to-center distance (Figure 3A–C and L).

**Figure 2—figure supplement 1.**
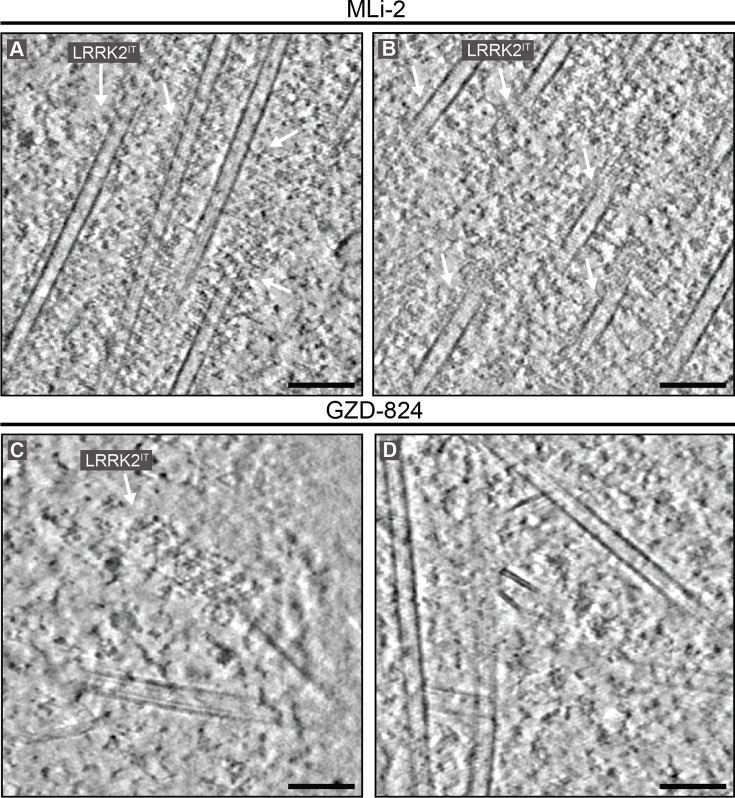
LRRK2^IT^ filament formation around microtubules is extensive when treated with type I, but not type II inhibitor. (**A–B**) Representative tomogram slice through the cytosol highlighting multiple LRRK2^IT^-decorated microtubules in a cell treated with MLi-2. (**C–D**) Tomogram snapshots of a cell treated with GZD-824 where the majority of microtubules are not decorated with LRRK2^IT^. Scale bars: A–D, 100 nm.

**Figure 2—figure supplement 2.**
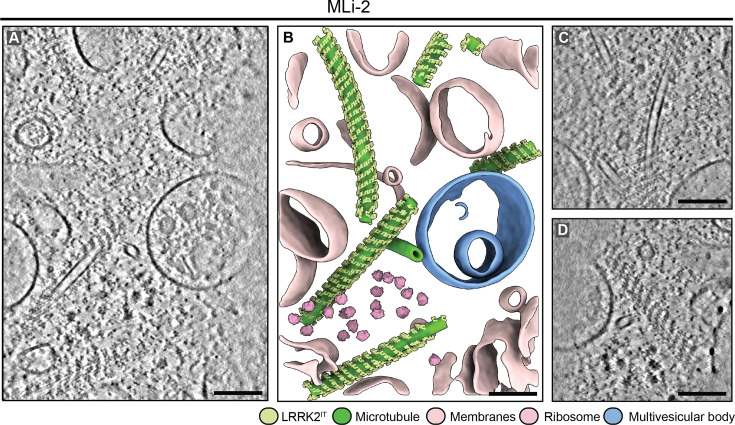
Type I inhibitor-treated cells have single microtubules decorated with LRRK2^IT^. (**A**) Tomogram slice of a cell treated with MLi-2 showing six single non-bundled microtubules decorated with LRRK2^IT^. (**B**) Segmentation of the tomogram shown in A highlighting LRRK2^IT^ filamentation around microtubules. (**C, D**) Tomogram snapshot of an LRRK2^IT^ lattice in MLi-2-treated cell. Lime green: LRRK2^IT^; green: microtubule; light pink: vesicles; blue: multivesicular bodies; pink: ribosomes. Scale bars: A–D, 100 nm.

**Figure 2—figure supplement 3.**
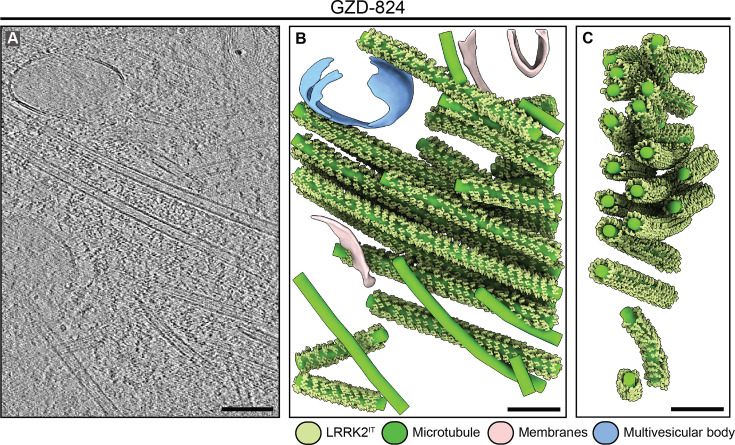
A single instance of LRRK2^IT^-decorated microtubules was captured in cells treated with type II inhibitor. (**A**) Tomogram snapshot of a cell treated with GZD-824 showing an LRRK2^IT^-decorated microtubule bundle. This is the single instance of LRRK2^IT^ bundle captured in cells treated with GZD-824. (**B**) Segmented representation of the tomogram shown in A. (**C**) Side view of the LRRK2^IT^-decorated microtubule bundle shown in B. Lime green: LRRK2^IT^; green: microtubule; light pink: vesicles; blue: multivesicular bodies. Scale bars: A–C, 100 nm.

**Figure 3—figure supplement 1.**
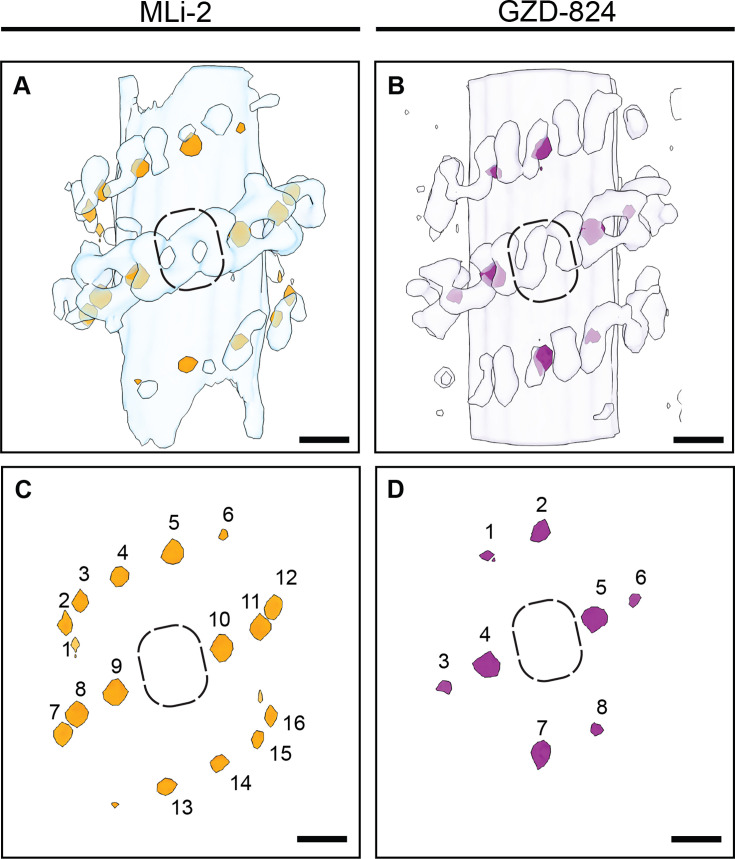
Nearest-neighbor analysis of a central LRRK2^IT^ subunit. (**A–B**) Nearest-neighbor analysis of a central LRRK2^IT^ subunit (highlighted in black) in MLi-2- and GZD-824-treated cells. (**C–D**) Annotation of immediate neighboring LRRK2^IT^ subunits within each lattice. In MLi-2-treated cells, the central LRRK2^IT^ subunit is surrounded by 16 nearest neighbors highlighted in orange. While in GZD-824-treated cells, the central LRRK2^IT^ subunit has only eight nearest neighbors shown in pink.

In contrast, tomograms acquired from type II GZD-824 inhibitor treatment resulted in reduced LRRK2 filamentation around microtubules, with only a few rare, isolated microtubules showing LRRK2^IT^ decoration (Figure 2E–H, Figure 2—figure supplement 1C–D). Interestingly, we captured a single bundle of LRRK2^IT^-decorated microtubules in the tomograms of GZD-824-treated cells (Figure 2—figure supplement 3). This bundle contained 15 microtubules with intermicrotubule spacing similar to LRRK2^IT^-decorated microtubule bundles in MLi-2-treated cells. Otherwise, fluorescence microscopy of GZD-824-treated cells showed LRRK2^IT^ was present as discrete cytosolic puncta, suggesting association with intracellular membranes (Figure 1F–G, Figure 1—figure supplement 1E–H, J). In these data, organelles appeared intact and morphologically unaltered, with no discernible densities or organized assemblies that could be confidently attributed to LRRK2^IT^ (Figure 1I). Aside from the absence of microtubule bundling, the overall ultrastructure of GZD-824-treated cells closely resembled that of MLi-2-treated and untreated cells (Watanabe et al., 2020).

### LRRK2^IT^ microtubule decoration is highly ordered in cells treated with type I compared to type II inhibitors

To characterize the differences between LRRK2^IT^ lattice organization in cells treated with different inhibitors, we performed subtomogram analysis of decorated microtubules and mapped the location of each individual LRRK2 dimer and quantified lattice parameters (Figure 3). In the MLi-2-treated cells, LRRK2^IT^ strands were organized around microtubules with a regularly spaced lattice, similar to the LRRK2^IT^ strands in cells not treated with the inhibitor (Figure 3A–E; Watanabe et al., 2020). Conversely, in GZD-824-treated cells, the LRRK2^IT^ lattice was rare, and when existent, it was disordered (Figure 3F–G). While average pitch, rise, and handedness of the filaments of the rare GZD-824-treated LRRK2 filaments were similar between the MLi-2 parameters (Figure 3H–K; Figure 3—figure supplement 1), the relative orientation of individual dimers was variable, and the lattice was highly irregular in GZD-824-treated cells (Figure 3F–G). Using nearest neighbor analysis, we found that in the LRRK2^IT^ lattice from MLi-2-treated cells, up to 16 close neighbors were placed with high precision, compared to only eight in the GZD-824-treated LRRK2^IT^ lattice (Figure 3J–K; Figure 1—figure supplement 1A–D). Altogether, this suggests that MLi-2 treatment stabilizes LRRK2 filamentation on microtubules, while GZD-824 disrupts LRRK2^IT^ lattice, weakening its association with microtubules.

### Subtomogram analysis of type I inhibitor-treated cells reveals the architecture of the N-terminal repeats of full-length LRRK2 in the closed-kinase conformation

Full-length LRRK2 structures with closed-kinase conformation have been determined, but the flexible N-terminal repeats remain disordered and are not resolved in these structures (Sanz Murillo et al., 2023; Chen et al., 2025; Zhu et al., 2024; Watanabe et al., 2020; Deniston et al., 2020; Snead et al., 2022; Myasnikov et al., 2021; Zhu et al., 2023; Martinez Fiesco et al., 2025). Given the stabilization of LRRK2^IT^ on microtubules with MLi-2 treatment, we attempted to resolve the full-length, closed-kinase conformation of LRRK2^IT^, focusing on resolving the N-terminal repeat architecture.

**Figure 4.**
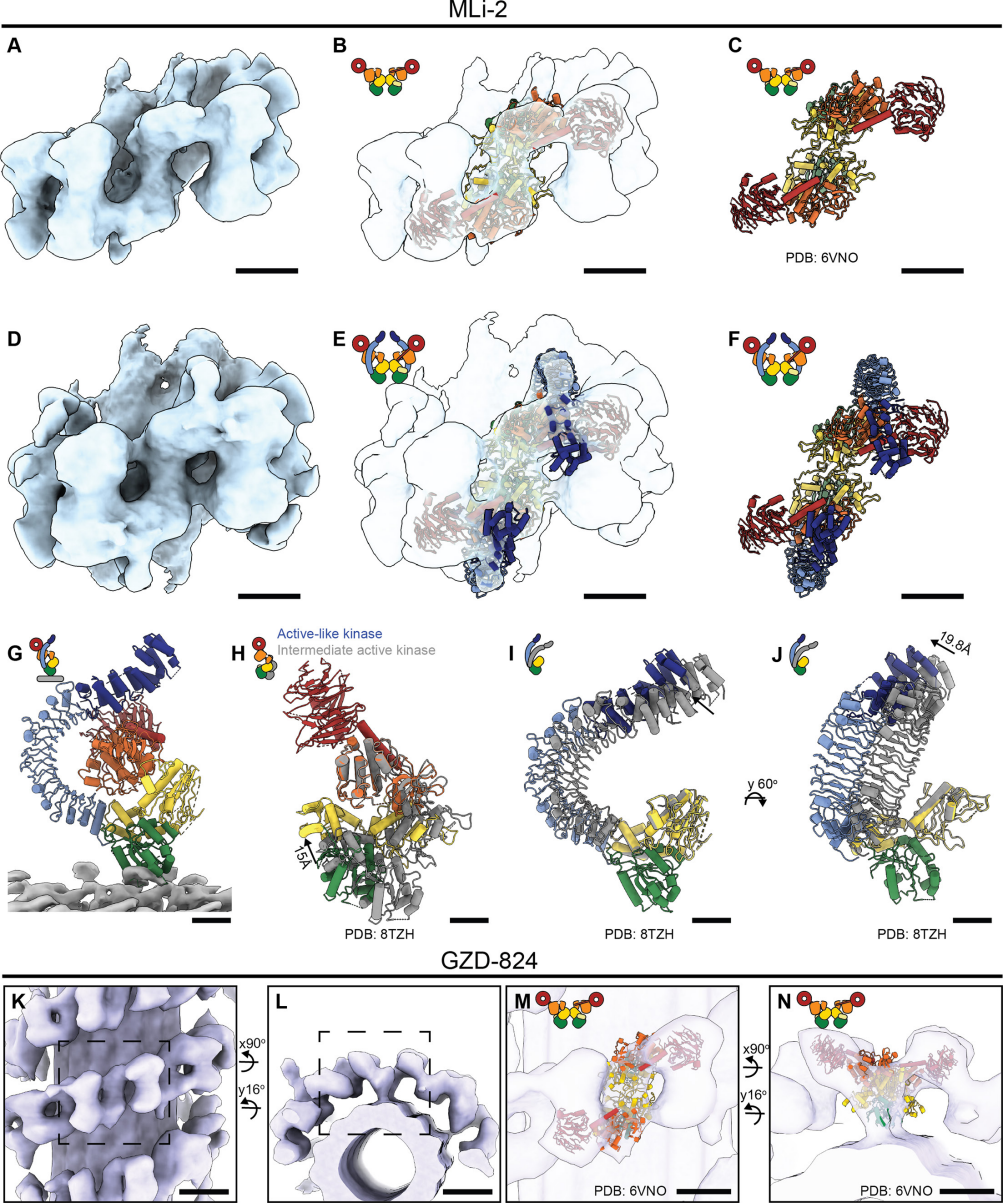
Subtomogram analysis of LRRK2 MLi-2 reveals structural details about the N-terminal domains of closed-kinase LRRK2^IT^. (**A**) Subtomogram average map of the microtubule-bound LRRK2 from MLi-2-treated cells (threshold value 0.24). (**B–C**) Molecular model of two central protomers of active kinase LRRK2 RCKW fit into map A. The protomers of LRRK2 fit well within the density corresponding to the active conformation of the LRRK2 kinase. The domains are colored according to Figure 1A. (**D**) Subtomogram average of LRRK2 displaying densities for the N-terminal domains emanating from the catalytic half of the protein (threshold value 0.14). (**E–F**) Molecular model of the two central protomers of full-length LRRK2 fit into the map shown in D. The model includes RCKW domains and N-terminal LRR-ANK domains. (**G**) Molecular model of a single subunit of full-length LRRK2 in active conformation interacting with a microtubule (gray) via ROC domain (green). (**H**) Comparison between the RCKW domains of LRRK2 in intermediate active kinase conformation (gray) and fully active full-length in-cell model of LRRK2 (colored). The kinase domain in the cellular map is fully closed, demonstrated by a 15 Å shift between the COR domains. (**I–J**) Comparison of the LRR and ARM domains of LRRK2 in the in-cell model (colored) versus the intermediate kinase-active LRRK2 structure (gray). The COR–ROC domains are aligned in both structures to highlight the positional shifts in the LRR and ARM domains (**K–L**). Subtomogram average of microtubule-bound LRRK2 from GZD-824-treated cells. (**M–N**) Molecular model of the active kinase RCKW domains fitted into the map shown in panel K. The molecular model aligns well with the active kinase conformation of LRRK2. The N-terminal domains are not visible in this map. Rotated views are shown. Scale bars: A–F, 5 nm; G–J, 2 nm; K–L, 10 nm; M–N, 5 nm.

First, we performed subtomogram analysis on regions of the microtubules that focused on an LRRK2^IT^ dimer and its surroundings (Figure 4). We focused refinements on the COR-COR dimerization interface, centering the reconstruction around four LRRK2 monomers, resulting in a 12 Å resolution map (map A; Figure 4A). We also refined the WD40-WD40 dimerization interface and focused refinements on four central subunits reaching 16 Å resolution (map B; Figure 4—figure supplement 1). These reconstructions allowed us to dock all the individual domains of LRRK2 into the map (Figure 4). To determine the conformation of the kinase domain of LRRK2^IT^, we fit the structures of active and inactive kinase of LRRK2 into map A (Figure 4A–C). Fitting the inactive kinase structure resulted in significant clashes between COR-COR domains of neighboring LRRK2^IT^ subunits. The closed-kinase structure of the catalytic domain fit well into map A (Figure 4A) with no clashes, confirming that the reconstruction represents LRRK2^IT^ in its closed-kinase conformation (Figure 4B–C). In maps A and B, we resolved a connection between an ROC domain and tubulin, present in the in vitro microtubule-associated LRRK2 RCKW structure, but previously elusive in the in situ maps (Figure 4G; Figure 4—figure supplement 1G–H; Deniston et al., 2020; Snead et al., 2022).

**Figure 4—figure supplement 1.**
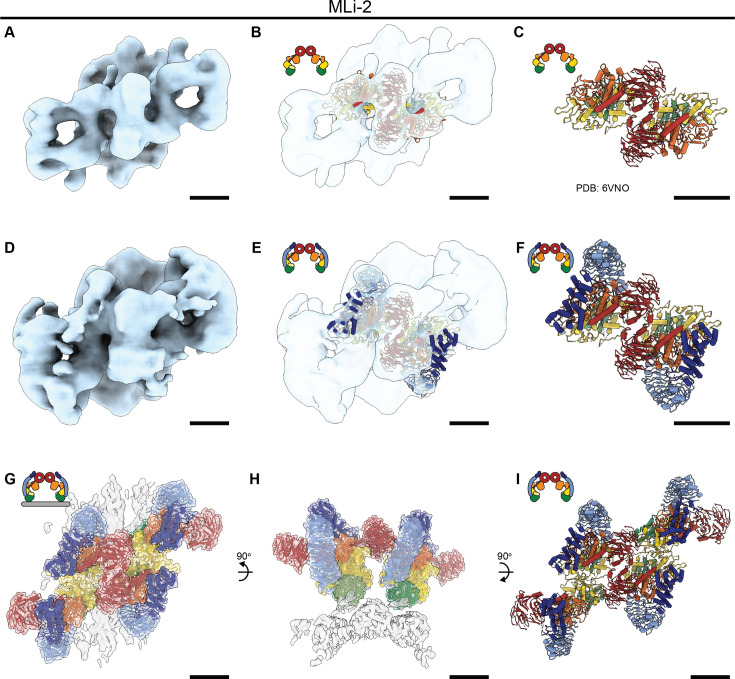
Subtomogram analysis of LRRK2^IT^ in MLi-2-treated cells centered on WD40-WD40 domain interaction interface. (**A**) Subtomogram average map of LRRK2^IT^ centered on the WD40-WD40 domain interface (threshold value: 0.12). (**B, C**) Molecular model of the closed-kinase LRRK2 fit in the map in A, demonstrating interaction interface between WD40-WD40 domains. Domain architecture color scheme is the same as given in Figure 1A. (**D**) Subtomogram average map at threshold value 0.24, showing the densities for N-terminal domains of LRRK2^IT^. (**E, F**) Molecular model of full-length LRRK2 fit in the map in D, showing the architecture of the N-terminal domains. (**G–I**) Molecular model of the four LRRK2 protomers fit into subtomogram map shown in D, demonstrating the overall architecture of the full-length LRRK2^IT^ assembly around a microtubule. Scale bars: A–I, 5 nm.

Notably, our maps have ordered density for the N-terminal LRR-ANK domains (Figure 4D–F), and thus we set out to build a full-length model of closed-kinase LRRK2^IT^. We based this model on our published structure of in vitro MLi-2-treated full-length LRRK2, where the kinase was captured in an intermediate activation state with the N-terminal repeats docked to the kinase, preventing it from adopting a fully active conformation (Sanz Murillo et al., 2023). We split this model into three parts: the WD40 and C-lobe of the kinase, the N-lobe of the kinase with ROC and COR domains, and the LRR and ANK domains. Each part was then aligned and fitted into map A (Figure 4D–F). Given the limited resolution of map A, we fit the model as three rigid bodies without flexible fitting or atomic refinement. When comparing this structure with the reference model of LRRK2^IT^ in its intermediate active conformation, we discerned key differences between the structures. First, the kinase was in the fully active conformation in the in situ map (Figure 4G–H). Second, the LRR-ANK domains were undocked from the kinase and WD40 domains and shifted by 19.8 Å as compared to the intermediate kinase-active structure (Figure 4I–J). In the closed-kinase form, the N-terminal repeats are known to undock from the catalytic core (Figure 4I–J), allowing easier access of LRRK2 substrates to the kinase domain. Here, we captured and modeled for the first time the N-terminal repeats undocked from the catalytic domains with kinase in an active-like conformation.

**Figure 5.**
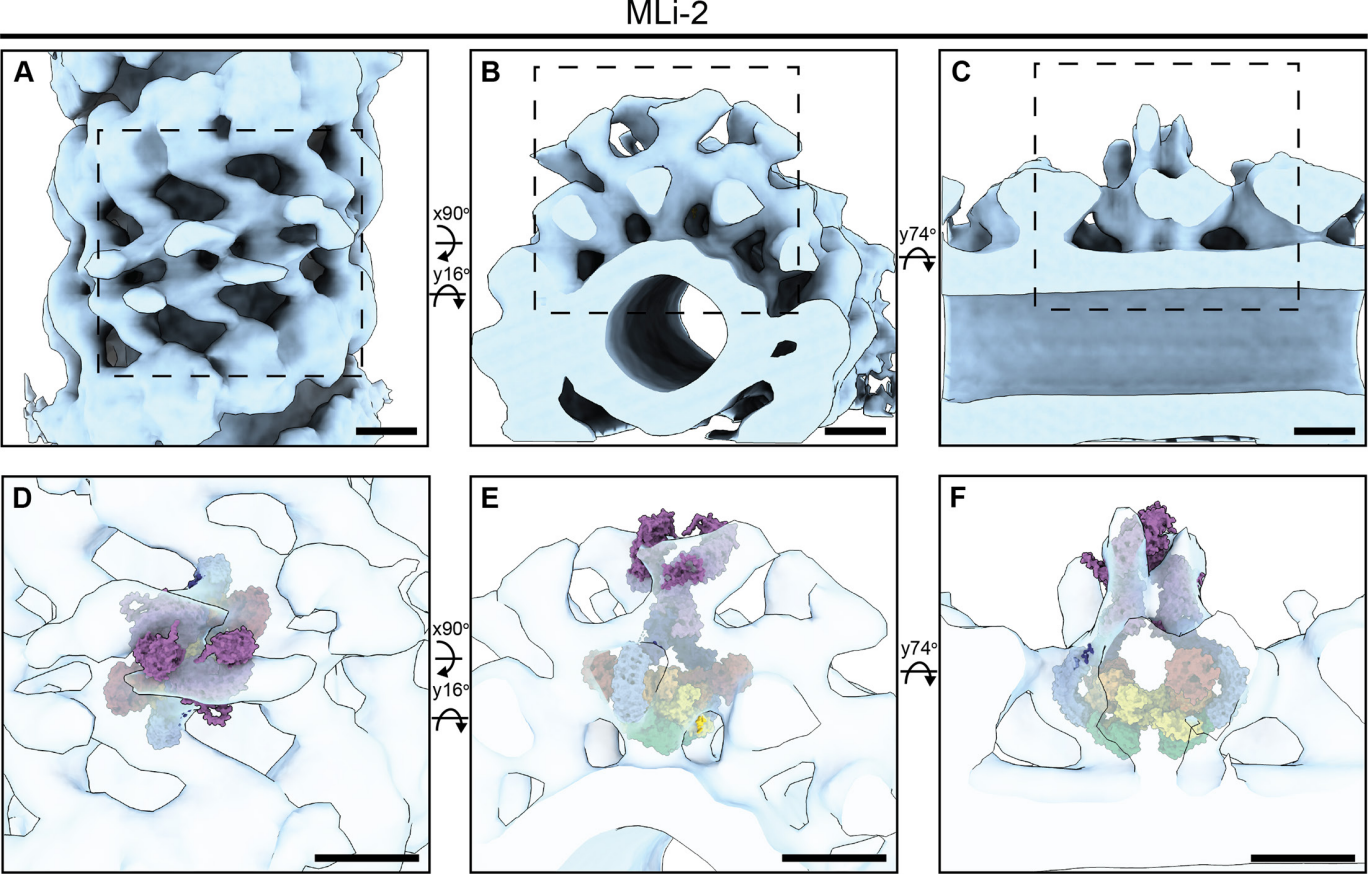
Model of full-length LRRK2^IT^ associated with microtubules in its active-like conformation. (**A**) Subtomogram average of LRRK2^IT^ lattice decorating a microtubule. (**B**) Cross-sectional view of the LRRK2^IT^ lattice. Catalytic domains are closest to the microtubule, while the outer layer represents the N-terminal protein-protein interaction domains of LRRK2. (**C**) Side view of the microtubule demonstrating the extension of the N-terminal layer of LRRK2. (**D–F**) Atomic model fit of full-length LRRK2^IT^ in the subtomogram average map. LRRK2 domain colors are as in Figure 1A. Scale bars: A–C, 10 nm; D–F, 10 nm.

Additionally, we performed subtomogram analysis in Dynamo on a larger LRRK2^IT^-decorated lattice that contained three layers of LRRK2^IT^ density around the microtubule; we refer to this average as map C. Refinement was focused on the central four LRRK2^IT^ subunits to better resolve additional protein densities within this larger lattice. In map C (Figure 5A; Figure 5—figure supplement 1), we discerned an additional layer of protein decoration that accommodated not only LRR and ANK but also the ARM domain of LRRK2^IT^ (Figure 5A–C; Figure 5—figure supplement 1). In this map, we first fit in our in situ model of LRRK2^IT^ and fit an AlphaFold2 model of the ARM domain into the additional densities (Figure 5D–F; Figure 5—figure supplement 1). While resolution of this map is moderate at 24.5 Å, densities that emanate from the catalytic part of the protein correspond to the LRR-ANK-ARM domains (Figure 5D–F; Figure 5—figure supplement 1; Figure 5—figure supplement 2). LRR-ANK-ARM domains all point outward, extending away from the catalytic domains of LRRK2^IT^ (Figure 5D–F). We hypothesize that the bundling visible in our maps could be reinforced by the connections between the LRRK2^IT^ N-terminal ARM domains between adjacent microtubules in a bundle (Figure 3A–C; Figure 5D–F).

**Figure 5—figure supplement 1.**
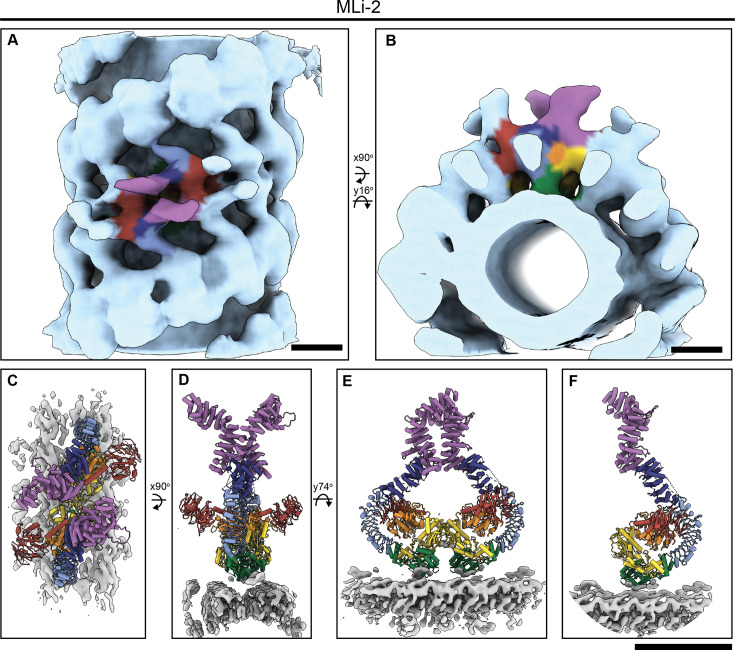
Model of full-length LRRK2^IT^ associated with microtubules in its active-like conformation. (**A–B**) Subtomogram average of LRRK2^IT^ lattice decorating a microtubule and a corresponding cross-section. (**C–E**) Molecular model of the closed-kinase LRRK2 fit in the map in A, demonstrating domain organization of the N-terminal LRR-ANK-ARM domains. (**F**) Molecular model of a single full-length LRRK2 fitted into the map shown in A, highlighting the architecture of the N-terminal domains. Domains are colored as in Figure 1A, and the microtubule is presented in gray (EMDB 25908). Scale bars: A–F, 10 nm.

**Figure 5—figure supplement 2.**
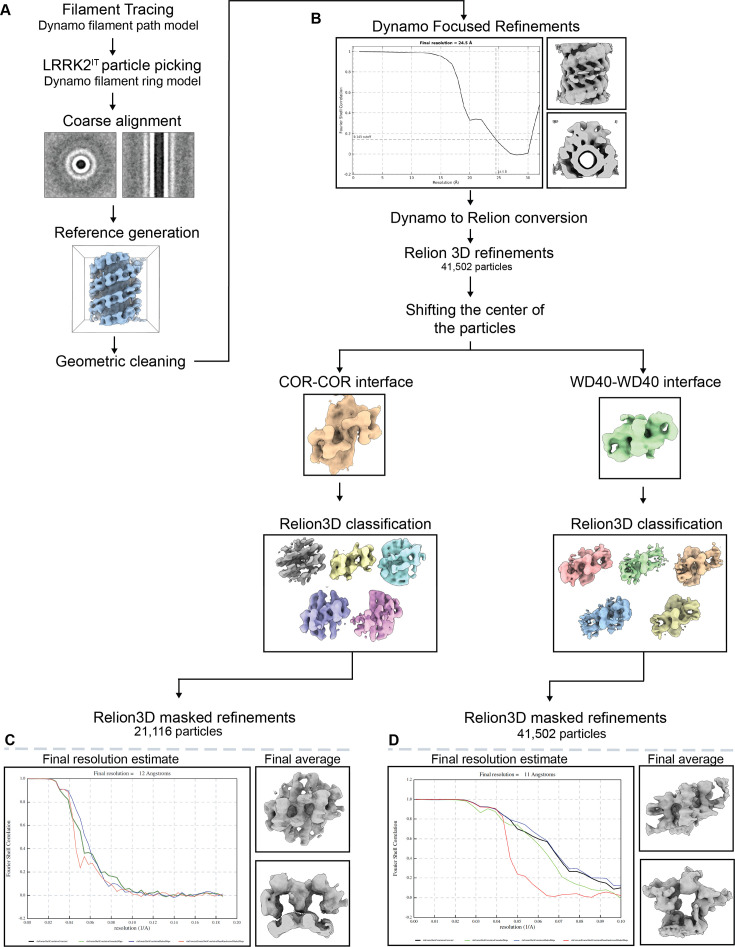
Data-processing workflow for LRRK2^IT^ in type I inhibitor-treated cells. (**A**) Cryo-electron tomography (cryo-ET) data processing workflow for LRRK2^IT^-decorated microtubules in MLi-2-treated cells. (**B**) Fourier shell correlation (FSC) curve for the Dynamo-refined MLi-2-treated LRRK2 subtomogram average presented in Figure 5. (**C**) Final maps and the gold-standard Fourier shell correlation (FSC) curves (0.143 cutoff) show the final resolution of the COR-COR domain interaction interface-centered LRRK2^IT^ refinement. (**D**) Final maps and the gold-standard FSC curves (0.143 cutoff) show the final resolution of the WD40-WD40 domain interaction interface-centered LRRK2^IT^ refinement.

Our attempts to characterize the molecular architecture of LRRK2^IT^ following treatment with GZD-824 showed minimal filamentation of LRRK2^IT^ on microtubules (Figure 2E–H). In tomograms of GZD-824-treated cells, only six individual LRRK2-decorated microtubules and a single LRRK2-decorated microtubule bundle were detected (Figure 2E–H, Figure 2—figure supplements 1–3). Subtomogram analysis revealed a disrupted LRRK2 strand geometry, compared to that observed in MLi-2-treated cells (Figure 3F–G). We obtained a subtomogram map of LRRK2^IT^ decoration at significantly lower resolution (~30 Å), in which only the RCKW domains could be resolved (Figure 4K–N). Fitting of the RCKW domains into this map was consistent with a closed-kinase conformation of LRRK2^IT^ (Figure 4M–N). A likely explanation for this observation is that GZD-824 treatment was insufficient to convert all pre-existing, microtubule-bound LRRK2 molecules from an active to an inactive kinase conformation. Instead, the residual filaments detected are composed of LRRK2 molecules that formed prior to inhibitor addition and are refractory to subsequent GZD-824 binding. This interpretation is consistent with the markedly reduced frequency of filamentation observed under GZD-824 treatment.

## Discussion

Utilizing cellular structural biology tools, we dissected the effects of different types of inhibitors on the localization and architecture of hyperactive LRRK2^IT^ expressed in 293T cells. In cells treated with type I MLi-2 inhibitor, we captured a significant number of microtubules with LRRK2^IT^ decoration, the majority of which formed extensive bundled networks of LRRK2^IT^ filaments (Figure 3). On the other hand, when cells were treated with GZD-824, only a few microtubules were decorated with LRRK2^IT^ (Figure 2E–H). Differences in LRRK2^IT^ propensity to form filaments can be explained by the distinct molecular mechanisms of action of inhibitor treatments. MLi-2 treatment is known to stabilize LRRK2 in its closed-kinase conformation, which in turn promotes filament formation around microtubules. These observations are in stark contrast to LRRK2 localization in cells treated with GZD-824, which stabilizes LRRK2’s kinase in its inactive conformation, weakening association with microtubules (Sanz Murillo et al., 2023; Zhu et al., 2024). These findings confirm that inhibitor-induced transitions of LRRK2^IT^ from its active to inactive kinase conformation modulate its interaction with microtubules, leading to a shift from extensive to minimal microtubule decoration in cells.

The physiological relevance of LRRK2 filamentation around microtubules remains controversial. Wild-type LRRK2 does not exhibit enriched microtubule localization, whereas several PD-associated mutants (e.g. I2020T, R1441H/G/S/T) show robust microtubule association. Microtubule decoration by LRRK2^IT^ has not been studied in cell types that endogenously express high levels of LRRK2, such as lung epithelial cells and brain-resident immune cells, including microglia and macrophages (Cook et al., 2017). Thus, it remains possible that aberrant LRRK2-microtubule interactions occur under physiological expression conditions, potentially disrupting homeostatic intracellular transport and being further exacerbated by type I LRRK2 inhibitors, as suggested by in vitro studies (Deniston et al., 2020; Twellsieck and Boecker, 2025).

In our previous work, we used in-cell cryo-ET to build an integrative model of the microtubule-associated catalytic ROC, COR, kinase, and WD40 (RCKW) domains of LRRK2 in its closed-kinase conformation (Watanabe et al., 2020). However, our model could not resolve the conformation of N-terminal LRR, ANK, and ARM domains. Full-length LRRK2 has been captured in vitro in its intermediate active conformation (Sanz Murillo et al., 2023; Chen et al., 2025; Zhu et al., 2024; Deniston et al., 2020; Snead et al., 2022; Myasnikov et al., 2021; Zhu et al., 2023). However, how the N-terminal repeats of LRRK2 are organized when the protein is in its closed-kinase conformation remained unresolved. Stabilization of LRRK2 in a closed-kinase conformation by MLi-2 treatment and microtubule association reduces conformational heterogeneity to permit structure determination of full-length LRRK2^IT^ with the N-terminal repeats undocked from the catalytic core. Therefore, the key novelty of this structure is that it captures full-length LRRK2^IT^ in a cellular, microtubule-associated closed-kinase state and shows that kinase closure is compatible with an undocked N-terminal architecture. This distinguishes the in situ closed-kinase state from previously described in vitro intermediate active states. This structure represents one stabilized closed-kinase state within a broader conformational ensemble of undocked N-terminal repeats.

Our results clarify the relationship between kinase conformation, repeat undocking, and microtubule association. Increased microtubule association observed for the I2020T mutant favors undocking of the N-terminal repeats, a prerequisite for kinase closure and filament assembly. In contrast, the G2019S mutation enhances catalytic activity without increasing microtubule association. Microtubule binding therefore reflects conformational state rather than kinase activity per se.

Together, these findings provide a structural view of full-length LRRK2 in a closed-kinase conformation and capture a resolved snapshot along its conformational continuum. Our results underscore how pathogenic mutations and distinct inhibitor classes reshape its structural landscape. More broadly, this work demonstrates the power of in-cell structural biology to illuminate dynamic regulatory transitions that are otherwise obscured by conformational heterogeneity.

## Methods

### Cell culture

HEK293FT cells were obtained from Thermo Fisher Scientific (Cat No R7007: RRIDCVCL_6911) and cells were cultured in Dulbecco’s Modified Eagle’s Medium (DMEM) formulated with high glucose. DMEM was supplemented by 10% (vol/vol) heat-inactivated fetal bovine serum (FBS), 0.1 mM Non-Essential Amino Acids (NEAA), 1 mM sodium pyruvate, 1% penicillin-streptomycin (Pen-Strep), and 500 μg/ml Geneticin Selective antibiotic. Cells were maintained at 37°C and 5% CO_2_, subcultured every 2–3 days at ~80% confluency. Cells were detached for subculture using 0.05% trypsin-EDTA. Cell line was tested for mycoplasma contamination and tested negative. The cell line identity was verified by ATCC using short tandem repeat (STR) profiling as part of their quality control procedures prior to distribution.

### Transfection experiments

293T cells were transfected using the Lipofectamine 3000 Transfection Kit (Thermo Fisher Scientific) according to the manufacturer’s protocol. For each transfection, 1 μg of GFP-tagged LRRK2 recombinant DNA was used (Addgene). Transfections were carried out in six-well plates. Cells were incubated for 48 hr post-transfection at 37°C and 5% CO_2_. Prior to sample preparation for fixation or cryo-EM grid preparation, cells were treated with 500 nM of MLi-2 for 2 hr, or 300 nM GZD-824 for 10–30 min. Following treatment, transfected cells were either fixed for fluorescence microscopy analysis or processed for cryo-ET grid preparation.

### Cryo-TEM grid preparation

Au Quantifoil R1/4 200 mesh grids were used to prepare cellular samples. Prior to cell deposition, the grids were patterned to enhance cell adhesion and positioning for cryo-ET (Sibert et al., 2021). In brief, grids were plasma-cleaned and were treated with PLL-PEG. Using UV laser, custom-defined micropatterns were created in the layer of PLL-PEG, creating adhesive regions using the PRIMO micropatterning system (Alveole). The patterned grids were incubated with fibronectin for 1 hr at room temperature before cells were deposited on them. Detailed protocol for patterning grids can be accessed here (Shaikh et al., 2023).

Cells were detached from culture dishes after transfection via trypsin treatment, counted, and approximately 200,000 cells were deposited on four grids in a 3 cm cell culture dish. The cells were then incubated on the patterned grids for 2 hr or until the cells were completely attached. Following attachment to the grids, samples were plunge-frozen using a manual custom plunger assembly. The freezing medium consisted of a 50/50 ethane/propane mixture. Grids were blotted for 6–8 s in a humidity-controlled room. Plunge-frozen grids were secured in autogrids and stored in liquid nitrogen until imaging.

### Cryo-FIB milling

Cryo-FIB milling was performed on an Aquilos 2+ system from Thermo Fisher Scientific. Scanning electron microscope images were taken using either 2 or 5 kV at a 30 pA beam current. The lamellae were prepared at an 11° stage angle. Milling was performed in a sequential manner starting from rough milling with 0.5–0.1 nA currents, followed by intermediate milling at 50–30 pA and final polishing using a 10 pA current. Target lamella thickness was 150–200 nm. A detailed protocol for cryo-FIB milling is described in previous publications (Lam and Villa, 2021; Wagner et al., 2020; Schaffer et al., 2015).

### Integrated cryo-FM

Cryo-FM was performed using an integrated fluorescence microscopy system on Aquilos 2+. The 20× air objective NA 0.7 was used for imaging. For GFP-tagged cellular samples, excitation at 470 nm was used for visualization. Illumination was set to 10% intensity with a 200 ms exposure time to minimize photodamage. Z-stacks spanning 10–20 μm in depth were acquired with step sizes ranging between 500 and 800 nm. This allowed imaging of the whole cellular volumes with adequate resolution and limited photodamage.

### Cryo-ET

TEM image acquisition was performed on a Titan Krios G3 system equipped with a K3 direct electron detector and Gatan energy filter. The milling slot of the autogrid was oriented perpendicular to the loading cartridge. Tilt-series were acquired using SerialEM software and Pacetomo-automated tilt-series acquisition methods (Mastronarde, 2005; Eisenstein et al., 2023). The images were collected at a target defocus range of 4–5 μm with a pixel size of 1.341 Å. Dose symmetric data acquisition schemes were implemented with a tilt range of ±54° with 3° increments (Hagen et al., 2017). The pre-tilt was calculated using Pacetomo, and acquisition was initiated from this position. A cumulative electron dose of approximately 140 e⁻/Å² was applied across the tilt-series.

### Tomogram reconstruction and annotation

Tilt-series were processed using a Warp-Imod pipeline (Tegunov et al., 2021; Tegunov and Cramer, 2019; Kremer et al., 1996). Initial preprocessing, including frame alignment, gain correction, and motion correction, was performed in Warp. Tilt-series were aligned in IMOD. The aligned tilt-series stacks were imported back into Warp 1.0.9/beta for 3D-CTF correction. Final tomograms were reconstructed in Warp and visualized in IMOD.

### Tomogram segmentation and visualization

Cellular membrane segmentation was performed using MemBrainSeg v0.0.9 with v10_alpha pretrained segmentation model, with manual clean-up performed in UCSF ChimeraX (Meng et al., 2023; Lamm et al., 2024). Microtubule coordinates were traced manually in IMOD and visualized in ChimeraX. Ribosomes were identified through template matching in Warp using an ab initio ribosome model, with subsequent coordinate refinement in Relion. LRRK2 subtomograms were extracted from Dynamo coordinate tables (Castaño-Díez et al., 2012). The segmented membranes, microtubules, LRRK2, and ribosome coordinates were inputted in ChimeraX and ArtiaX for comprehensive visualization (Ermel et al., 2022).

### Microtubule tracing and protofilament number analysis

Tomograms were visualized in IMOD to identify LRRK2^IT^-decorated microtubules. These microtubules were manually traced in IMOD and coordinates were saved as contours. Filaments were cropped equidistantly along the traced microtubule coordinates using the filament path function in Dynamo. The cropped volumes were averaged and smoothed to generate an ab initio reference for a microtubule. Based on this ab initio reference, a cylindrical featureless reference was generated. Each microtubule was cropped, and alignment was run to symmetrize each individual microtubule. The results of the alignment were evaluated to assess the protofilament number of the microtubule.

### LRRK2 subtomogram analysis

LRRK2-decorated microtubule coordinates were used to extract subtomograms of LRRK2 using the Dynamo filament path function. 15 subunits at 8A separation were used to crop the LRRK2 subtomograms along the microtubule path. Cropped particles were averaged to generate the initial reference. A spherical mask around the central four subunits of LRRK2 was generated and initial refinements were run in Dynamo to align cropped particles. We removed duplicate particles from these subtomograms and exported them for import into Relion3. The subtomograms were refined in Relion3 with a C1 symmetry (Bharat and Scheres, 2016). Focused refinements were performed on the four central subunits of LRRK2^IT^. 3D classification was performed following initial refinements, and classes with distinct LRRK2 decorations were selected for further refinements. The refinements were either centered on the COR-COR interface of the protein or the WD40-WD40 interaction interface. The center of the box was shifted accordingly, and refinements were performed with newly extracted particles.

### Model building

Structures of previously published LRRK2 were used as references for model building. ChimeraX rigid body fitting was used to fit each individual domain into the density maps. The fitting was performed stepwise. The domains of WD40 and kinase C-lobe were separated and fit into the maps together. Following this, kinase N-lobe, COR, and ROC domains were fitted into the density for the LRRK2. LRR-ANK domains were fit separately as well, accounting for the remaining electron density in the in situ maps. ARM domain was obtained through AlphaFold2 model of LRRK2 and separated from the rest of the protein. ARM domain was individually mapped into the density for the N-terminal domains in electron density maps with larger lattice of LRRK2 accounting for the extra densities.

## Data Availability

Cryo-EM density maps have been deposited in the Electron Microscopy Data Bank under accession numbers EMD-77327 for Map A, corresponding to the focused map of the COR:COR interaction interface in LRRK2I2020T + MLi-2 + microtubules; EMD-77328 for Map B, corresponding to the focused map of the WD40:WD40 interaction interface in LRRK2I2020T + MLi-2 + microtubules; EMD-77329 for the large-lattice map of LRRK2I2020T + MLi-2 + microtubules; and EMD-77330 for the LRRK2I2020T + GZD-824 + microtubule map. The atomic model rigid-body docked into our maps is available under PDB accession code 8TZH. The following datasets were generated: BasiashviliT VillaE 2026Focused map of the COR:COR interaction interface in microtubule-associated LRRK2 I2020T filaments formed in the presence of MLi-2EMDataResourceEMD-77327 BasiashviliT VillaE 2026LRRK2 I2020T filaments formed in the presence of MLi-2EMDataResourceEMD-77328 BasiashviliT VillaE 2026Focused map of four central LRRK2 I2020T units in the microtubule-associated filament lattice formed in the presence of MLi-2EMDataResourceEMD-77329 BasiashviliT VillaE 2026Microtubule-associated LRRK2 I2020T filaments in HEK293T cells treated with GZD-824EMDataResourceEMD-77330 Sanz-MurilloM Villagran-SuarezA Alegrio LouroJ LeschzinerA 2023Structure of full-length LRRK2 bound to MLi-2 (I2020T mutant)Worldwide Protein Data Bank10.2210/pdb8tzh/pdb

## Additional files

Supplementary file 1. Supplementary Materials: Materials Used and Data Acquisition Parameters. Table A. List of key reagents and tools used to perform this study. Table B. Data acquisition table for cryo-electron tomography (cryo-ET) datasets used in this study.

MDAR checklist
