# Supplementary material for: In-cell cryo-electron tomography reveals differential effects of type I and type II kinase inhibitors on LRRK2 filament formation and microtubule association
Source: eLife. 2026 Jul 27;15:RP111075. doi: 10.7554/eLife.111075 (PMC13405622; doi:10.7554/eLife.111075)
Supplement: Supplementary file 1. — Table A. List of key reagents and tools used to perform this study. Table B. Data acquisition table for cryo-electron tomography (cryo-ET) datasets used in this study. [file elife-111075-supp1.docx]

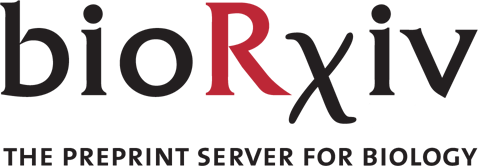


Supplementary File 1

**In-cell cryo-electron tomography reveals differential effects of type I and type II kinase inhibitors on LRRK2 filament formation and microtubule association**

T. Basiashvili, J. Hutchings, S. Chen, E. P. Karasmanis, W. A. Flaherty, A. E. Leschziner, E. Villa

* Co-Corresponding authors: A.L. (aleschziner@ucsd.edu) E.V. (evilla@ucsd.edu)

**Tables A-B**

### **Table A. List of key reagents and tools used to perform this study.**

| Reagent or Resource | Source | Catalog Number / Identifier |
| --- | --- | --- |
| Chemicals, Peptides, and Recombinant proteins | | |
| MLi-2 | Tocris | 5756 |
| Taxol (paclitaxel) | Cell Signaling Technology | 9807 |
| GZD-824 | Cayman Chemical | 21508 |
| DNL201 (GNE0877) | MedChem Express | HY-15796 |
| Poly-L-Lysine solution 0.1% (w/v) in H_2_O (PLL-PEG) | Millipore sigma | P8920 |
| Fibronectin | Thermo Fisher Scientific (Gibco) | 33016015 |
| Fetal Bovine Serum (FBS) | Thermo Fisher Scientific (Gibco) | A5209402 |
| MEM NON-Essential Amino Acis (NEAA) | Thermo Fisher Scientific (Gibco) | 11140050 |
| Geneticin | Thermo Fisher Scientific (Gibco) | 10131035 |
| Pen-Strep | Thermo Fisher Scientific (Gibco) | 15140122 |
| Sodium Pyruvate | Thermo Fisher Scientific (Gibco) | 11360070 |
| DMEM (high-glucose) | Thermo Fisher Scientific (Gibco) | 11965092 |
| DMSO, Anhydrous | Thermo Fisher Scientific | D12345 |
| Trypsin-EDTA 0.25% | Thermo Fisher Scientific | 25200056 |
| Critical Commercial Assays | | |
| Lipofectamine 3000 Transfection Reagent | Thermo Fisher Scientific | L3000001 |
| Deposited Data | | |
|  |  |  |
| Cell Line | | |
| HEK293FT cells | Thermo Fisher Scientific | R70007 |
| Recombinant DNA | | |
| GFP-LRRK2-WT | Addgene | 25044 |
| GFP-LRRK2-I2020T | Watanabe et al. 2021 |  |
| GFP-LRRK2-G2019S | Addgene | 25045 |
| Software and Algorithms | | |
| TFS TUI for Aquilos | Thermo Fisher Scientific | - |
| MAPS 3.24 | Thermo Fisher Scientific | - |
| iFLM V2 | Thermo Fisher Scientific | - |
| Serial EM | Mastronarde, 2005 | - |
| Pace-Tomo | Eisenstein et al., 2023 | - |
| Warp 1.0.9 / beta 1 | Tegunov et al., 2021, Tegunov et al., 2019 | - |
| IMOD | Kremer et al., 1996 | - |
| MemBrainSeg | Lamm et al., 2024 | - |
| ChimeraX | Meng et al., 2023 | - |
| ArtiaX | Ermel et al., 2022 | - |
| DYNAMO | Castano-Diez et al., 2012 | - |
| RELION | Bharat et al., 2015 | - |
| Other | | |
| Au Quantifoil R1/4 grids 200 mesh | Quantifoil Micro tools | NA |
| #1 Whatman filter paper | Whatman | 1001 |
| Liquid Nitrogen | Airgas | - |
| Ethane-Propane mixture | Airgas | - |

### **Table B. Data acquisition table for cryo-ET datasets used in this study.**

| Parameters | Data # MLi-2 | Data # GZD-824 |
| --- | --- | --- |
| Magnification | 63,000 | 63,000 |
| Voltage (kV) | 300 kV | 300 kV |
| Total dose (e⁻/Å²) | ~140 e⁻/Å² | ~140 e⁻/Å² |
| Defocus range (μm) | -2 to -5 | -2 to -5 |
| Acquisition scheme | Dose symmetric | Dose symmetric |
| Pixel size (Å) | 1.3410 Å | 1.3410 Å |
| No. of frames | 6 | 6 |
| # of tomograms | 26 | 6 |
| # of LRRK2^IT^ decorated microtubules | 254 | 27 |
| Final particle number | 21,116 particles | 6,212 particles |
| Symmetry imposed | C2 | C1 |
| Map resolution (Å) | ~12 Å | ~30 Å |
