# Supplementary material for: In-cell cryo-electron tomography reveals differential effects of type I and type II kinase inhibitors on LRRK2 filament formation and microtubule association
Source: eLife. 2026 Jul 27;15:RP111075. doi: 10.7554/eLife.111075 (PMC13405622; doi:10.7554/eLife.111075)
Supplement: Peer review history

# Editor report: eLife Assessment

**DOI:** 10.7554/eLife.111075.3.sa0

**Authors:** Sjors HW Scheres

**Affiliations:** MRC Laboratory of Molecular Biology United Kingdom

**Keywords:** Convincing, Important

This study reports **important** findings by showing that two classes of kinase inhibitors, which stabilise the LRRK2 enzyme in either an active (Type I) or inactive state (Type II), have distinct effects on the formation of LRRK2 filaments and their association with cellular structures. Using correlative light microscopy, cryo-electron tomography and sub-tomogram averaging, the authors provide **convincing** evidence that a Type I inhibitor leads to the extensive decoration of microtubules with LRRK2 in a closed-kinase conformation, and that such decoration is not seen for a type-II inhibitor. The conclusions are consistent with previous work, although the physiological relevance of the work remains somewhat limited due to reliance on overexpression and the use of a rare mutation in a single cell type.

# Referee report: Reviewer #1 (Public review)

**DOI:** 10.7554/eLife.111075.3.sa1

[Editors' note: Given the minor nature of this revision, the editors have not sent this back to the original reviewers. The original reviews have been included.]

In this study, the authors set out to determine how two classes of kinase inhibitors, which stabilise a disease-relevant enzyme in either an active (Type I) or inactive state (Type II), influence its organisation and interactions with microtubule filaments in cells. Using the state-of-the-art in-cell structural imaging approaches, they examine how these compounds affect the formation of protein filaments and their association with microtubules, and succeed in defining the underlying structural basis for these differences.

A major strength of the work is the application of in-cell cryo-electron tomography combined with correlative imaging, which enables direct visualisation of protein organisation in a near-native cellular context. The data convincingly demonstrate that the Type I inhibitor compound stabilising the active state promotes extensive LRRK2 filament formation and microtubule bundling, whereas compounds stabilising the inactive state markedly reduce these interactions. The structural analysis further provides insight into how conformational states relate to filament organisation, including modelling of previously unresolved regions of the protein.

These findings are internally consistent and align well with prior biochemical and structural studies, many of which were performed by the same team.

There are, however, some limitations that should be noted. The experiments rely on overexpression of the I2020T mutant form of the LRRK2 protein, which is a rare variant, in a single cell type (293T cells), which may not fully reflect endogenous behaviour or wild-type LRRK2 in a physiological context. In addition, while the imaging data are compelling, the functional consequences of the observed filament formation and microtubule association remain unclear.

The study therefore provides strong descriptive and structural insight, but more limited evidence linking these observations to cellular or disease-relevant outcomes.

Overall, the authors largely achieve their aims, and the results support their central conclusion that different classes of kinase inhibitors have distinct effects on protein organisation in cells. The work represents an important advance in understanding how small molecules can reshape protein architecture in a cellular environment, with potential implications for therapeutic strategies. The methodological approach will also be of broad interest to the field, as it highlights the power of in-cell structural biology to study dynamic protein assemblies that are difficult to capture using traditional approaches.

# Referee report: Reviewer #2 (Public review)

**DOI:** 10.7554/eLife.111075.3.sa2

Summary:

Mutations in Leucine-Rich Repeat Kinase 2 (LRRK2) are a major cause of Parkinson's disease. LRRK2 PD-related mutations all result in increased kinase activity. Therefore, LRRK2 has been the focus of the development of kinase inhibitors. So far, two classes of kinase inhibitors have been identified: type 1 LRRK2-specific inhibitors that stabilize LRRK2 in a closed active-like conformation and broad-range type 2 inhibitors that stabilize LRRK2 in an open inactive-like conformation. Basiashvili et al. used here in cell structural biology to study the effect of both type 1 and type 2 inhibitors on the localization and structural conformation of LRRK2-I2020T.

Strengths:

They showed that Type 1 and not Type 2 inhibitors induce LRRK2 filament/ on microtubules. Furthermore, they were able to build a structural map of full-length LRRK2 I2020T bound to a Type 1 inhibitor in a closed kinase confirmation. Together, this work thus confirms the data of previous studies that showed that LRRK2 Type 1 and 2 inhibitors differently affect filament formation.

Previous Weaknesses:

All conclusions are fully supported by the provided data. However, as the authors indicated themselves, the physiological relevance of LRRK2 microtubule binding is questionable. Furthermore, although the authors used a full-length LRRK2 protein, like in previously published structures, the resolution of the N-terminal domains is rather poor. Therefore, it also remains unclear what we learn from this structure compared to the previously published structures.

# Referee report: Reviewer #3 (Public review)

**DOI:** 10.7554/eLife.111075.3.sa3

Summary:

This paper describes new insights into the effects of type-I and type-II LRRK2 inhibitors on HEK293T cells that over-express GFP-labeled LRRK2-I2020T. Using correlative light microscopy and cryo-electron tomography, a type-I inhibitor leads to the extensive decoration of microtubules with LRRK2, which is not seen for a type-II inhibitor. Subtomogram averaging reveals that LRRK2 binds to the microtubules in a closed-kinase conformation, with density for the N-terminal arms.

Strengths:

The paper is well written; the CLEM and cryo-ET appear to be done to a high standard. Consequently, I have only minor comments.

Weaknesses:

The resolution of the subtomogram averages is somewhat limited, but the authors have adequately limited the number of degrees of freedom in the fitting of their atomic models by only allowing rigid-body transformations of separate parts of LRRK2.

The authors should include FSC curves between the rigid-body fitted atomic models and the various sub-tomogram average maps.

Comment on the current version from the Reviewing Editor:

I do note that Ext Data Fig 8 does not yet contains the requested model-vs-map FSC curves. I guess this is an oversight and trust that the authors will remedy this during the production process. They might also want to explain what the black, red, green and blue FSC curves are in the current figure or only show the black (solvent-corrected FSC) curve, together with the requested model-vs-map curve.

# Author comment: Author response

**DOI:** 10.7554/eLife.111075.3.sa4

**Authors:** Tamar Basiashvili, Joshua Hutchings, Siyu Chen, Eva P Karasmanis, William Alexander Flaherty, Andres E Leschziner, Elizabeth Villa

**Affiliations:** University of California, San Diego La Jolla United States; University of California, San Diego La Jolla United States; University of California, San Diego La Jolla United States; University of California, San Diego San Diego United States; University of California, San Diego La Jolla United States; Aligning Science Across Parkinson's (ASAP) Collaborative Research Network, Chevy Chase, MD 20815, USA; Department of Cellular and Molecular Medicine, School of Medicine, University of California San Diego, La Jolla, CA 92093, USA San Diego United States; University of California, San Diego La Jolla United States

The following is the authors’ response to the original reviews.

**Public Reviews:**

**Reviewer #1 (Public review):**

In this study, the authors set out to determine how two classes of kinase inhibitors, which stabilise a disease-relevant enzyme in either an active (Type I) or inactive state (Type II), influence its organisation and interactions with microtubule filaments in cells. Using the state-ofthe-art in-cell structural imaging approaches, they examine how these compounds affect the formation of protein filaments and their association with microtubules, and succeed in defining the underlying structural basis for these differences.

A major strength of the work is the application of in-cell cryo-electron tomography combined with correlative imaging, which enables direct visualisation of protein organisation in a near-native cellular context. The data convincingly demonstrate that the Type I inhibitor compound stabilising the active state promotes extensive LRRK2 filament formation and microtubule bundling, whereas compounds stabilising the inactive state markedly reduce these interactions. The structural analysis further provides insight into how conformational states relate to filament organisation, including modelling of previously unresolved regions of the protein.

These findings are internally consistent and align well with prior biochemical and structural studies, many of which were performed by the same team.

There are, however, some limitations that should be noted. The experiments rely on overexpression of the I2020T mutant form of the LRRK2 protein, which is a rare variant, in a single cell type (293T cells), which may not fully reflect endogenous behaviour or wild-type LRRK2 in a physiological context. In addition, while the imaging data are compelling, the functional consequences of the observed filament formation and microtubule association remain unclear.

The study therefore provides strong descriptive and structural insight, but more limited evidence linking these observations to cellular or disease-relevant outcomes.

Overall, the authors largely achieve their aims, and the results support their central conclusion that different classes of kinase inhibitors have distinct effects on protein organisation in cells. The work represents an important advance in understanding how small molecules can reshape protein architecture in a cellular environment, with potential implications for therapeutic strategies. The methodological approach will also be of broad interest to the field, as it highlights the power of in-cell structural biology to study dynamic protein assemblies that are difficult to capture using traditional approaches.

We thank the reviewer for their thoughtful and positive assessment of our work. We appreciate their recognition that in-cell cryo-electron tomography and correlative imaging provide a powerful approach for directly visualizing how small-molecule inhibitors reshape LRRK2 organization in a cellular environment.

We agree that the use of overexpressed LRRK2I2020T in HEK293T cells represents an important limitation of the present study. This experimental system was selected because it enabled visualization and structural analysis of inhibitor-dependent LRRK2 assemblies in cells. However, the extent to which these observations apply to endogenous LRRK2, wild-type protein, other disease-associated variants, or physiologically relevant cell types remains to be established.

We also agree that the functional consequences of inhibitor-dependent LRRK2 filament formation and microtubule association remain unresolved. The goal of the present study was to define how type I and type II kinase inhibitors alter the cellular organization and structural state of LRRK2. Our data demonstrate that these inhibitor classes have markedly different effects on LRRK2 filament formation and microtubule association in cells, and provide a structural framework for understanding these differences. Future studies will be required to determine how these assemblies influence LRRK2 signaling, microtubule-based processes, and diseaserelevant cellular phenotypes.

We thank the reviewer for highlighting both the methodological significance of this work and its potential implications for understanding how therapeutic molecules remodel protein architecture in cells.

**Reviewer #2 (Public review):**

Summary:

Mutations in Leucine-Rich Repeat Kinase 2 (LRRK2) are a major cause of Parkinson's disease. LRRK2 PD-related mutations all result in increased kinase activity. Therefore, LRRK2 has been the focus of the development of kinase inhibitors. So far, two classes of kinase inhibitors have been identified: type 1 LRRK2-specific inhibitors that stabilize LRRK2 in a closed active-like conformation and broad-range type 2 inhibitors that stabilize LRRK2 in an open inactive-like conformation. Basiashvili et al. used here in cell structural biology to study the effect of both type 1 and type 2 inhibitors on the localization and structural conformation of LRRK2-I2020T.

Strengths:

They showed that Type 1 and not Type 2 inhibitors induce LRRK2 filament/ on microtubules.

Furthermore, they were able to build a structural map of full-length LRRK2 I2020T bound to a Type 1 inhibitor in a closed kinase confirmation. Together, this work thus confirms the data of previous studies that showed that LRRK2 Type 1 and 2 inhibitors differently affect filament formation.

Weaknesses:

All conclusions are fully supported by the provided data. However, as the authors indicated themselves, the physiological relevance of LRRK2 microtubule binding is questionable. Furthermore, although the authors used a full-length LRRK2 protein, like in previously published structures, the resolution of the N-terminal domains is rather poor. Therefore, it also remains unclear what we learn from this structure compared to the previously published structures.

We thank the reviewer for their positive evaluation of our study and for recognizing that our conclusions are supported by the data.

We agree that the physiological relevance of LRRK2 filament formation and microtubule association remains an important open question. Our study was designed to determine how type I and type II inhibitors affect the cellular organization and structural conformation of LRRK2. We explicitly acknowledge that future studies using endogenous LRRK2, disease-relevant cellular systems, and functional assays will be necessary to determine the biological significance of inhibitor-induced microtubule association.

We also appreciate the reviewer’s comment regarding the resolution of the N-terminal domains. Although the N-terminal density does not support detailed atomic interpretation, its visualization provides information about the global organization of full-length LRRK2 within an inhibitorinduced, microtubule-associated assembly in cells. Importantly, our study does not claim highresolution structural determination of the N-terminal regions. Rather, the advance is the in-cell structural observation of full-length LRRK2^I2020T^ in a type I inhibitor-stabilized, closed-kinase conformation, together with density indicating that the N-terminal repeat regions adopt an organization within the microtubule-associated lattice.

We have revised the manuscript to clarify this point and to more carefully distinguish the structural information supported by the density from interpretations that would require higherresolution data.

**Reviewer #3 (Public review):**

Summary:

This paper describes new insights into the effects of type-I and type-II LRRK2 inhibitors on HEK293T cells that over-express GFP-labeled LRRK2-I2020T. Using correlative light microscopy and cryo-electron tomography, a type-I inhibitor leads to the extensive decoration of microtubules with LRRK2, which is not seen for a type-II inhibitor. Subtomogram averaging reveals that LRRK2 binds to the microtubules in a closed-kinase conformation, with density for the N-terminal arms.

Strengths:

The paper is well written; the CLEM and cryo-ET appear to be done to a high standard. Consequently, I have only minor comments.

Weaknesses:

The resolution of the subtomogram averages is somewhat limited, but the authors have adequately limited the number of degrees of freedom in the fitting of their atomic models by only allowing rigid-body transformations of separate parts of LRRK2.

The authors should include FSC curves between the rigid-body fitted atomic models and the various sub-tomogram average maps.

We thank the reviewer for their positive assessment of the manuscript and for recognizing the quality of the correlative imaging and in-cell cryo-electron tomography analyses.

We also appreciate the reviewer’s recognition that our interpretation of the maps was appropriately constrained by fitting domains as rigid bodies, rather than attempting unsupported high-resolution model refinement.

We thank the reviewer for highlighting this and apologize for the oversight. We have added all the missing FSC curve plots of subtomogram maps presented in this study in Extended Data Figure 8.

**Recommendations for the authors:**

Reviewer #1 (Recommendations for the authors):

I think the current study is OK as it is, and the authors have taken this as far as they can.

In future work, for either the authors or others in the field, it will be important to determine whether endogenous LRRK2 can be recruited to microtubules in response to compounds that stabilise the active state, particularly in cell types that are more relevant to Parkinson's disease. Does this cause a roadblock that impacts microtubule-driven transport? Establishing whether such recruitment occurs under physiological expression levels will be critical for assessing the broader relevance of the findings.

In addition, it would be valuable to evaluate whether these Type 1 compounds have detrimental cellular effects linked to altered endogenous LRRK2-driven microtubule association, and whether inhibitors that stabilise the inactive state offer a potential advantage by avoiding this phenotype.

We thank the reviewer for insightful recommendations for future studies.

**Reviewer #2 (Recommendations for the authors):**

(1) Figure 5: What is map C, and how is it different from the other maps? The authors indicate that the resolution of the N-terminal domains is moderate. How certain are the authors of the fit of these domains? Since map C is not provided in the supplemental, it is not possible to check this.

We apologize for this oversight. We have updated the text to reflect how the map C was calculated. Now the text reads:

“Additionally, we performed subtomogram analysis in Dynamo on a larger LRRK2^IT^decorated lattice that contained three layers of LRRK2^IT^ density around the microtubule; we refer to this average as map C. Refinement was focused on the central four LRRK2^IT^ subunits to better resolve additional protein densities within this larger lattice. In map C (Fig. 5A; Ext. Fig. 7).”

In addition, we updated the figure 5D-F to demonstrate clear fit of the N-terminal domains into the presented map. We also added an Extended Data Figure 7 to the supplemental materials to highlight the fit of the model in the map and highlight the areas that would correspond to the Nterminal domains of LRRK2. We hope these updates demonstrate a good fit and justify observations highlighted in the paper.

(2) The authors convincingly confirm that LRRK2 Type 1 and 2 inhibitors differently affect filament formation and that type 1 LRRK2-specific inhibitors stabilize LRRK2 in a closed activelike conformation. However, from the way the paper is written, it is unclear what we learn from this new structural data. How similar is the current structure compared to the previous structures? What is the novelty?

We thank the reviewer for noting that this is unclear and giving us the opportunity to highlight it in the manuscript. We have added the following sentence in the discussion:

“However, how the N-terminal repeats of LRRK2 are organized when the protein is in its closedkinase conformation remained unresolved. Stabilization of LRRK2 in a closed-kinase conformation by MLi-2 treatment and microtubule association reduces conformational heterogeneity to permit structure determination of full-length LRRK2^IT^ with the N-terminal repeats undocked from the catalytic core. Therefore, the key novelty of this structure is that it captures full-length LRRK2^IT^ in a cellular, microtubule-associated closed-kinase state and shows that kinase closure is compatible with an undocked N-terminal architecture. This distinguishes the in situ closed-kinase state from previously described in vitro intermediate active states.”

Minor comments:

(1) "Its C-terminal catalytic region is composed of WD40, Roc GTPase, Kinase and COR (RCKW) domains."

Suggest changing this to Roc GTPase, Cor, Kinase and WD40 (RCKW) domains for clarity/following of abbreviation.

We have made this change.

(2) "In the MLi-2 treated cells, LRRK2IT strands were organized around microtubules with a regularly spaced lattice, similar to the LRRK2IT strands in cells not treated without the inhibitor (Fig. 3A-E)"

Phrasing, correct the underlined portion.

We have made this change.

(3) "While average pitch. rise, and handedness of the filaments of the rate GZD-824 treated LRRK2 filaments were similar..."

Punctuation.

We have made this change.

(4) "Our results clarify the relationship between kinase conformation, repeat undocking, and microtubule association. Increased microtubule association observed for I2020T mutant favors repeat undocking, a prerequisite for kinase closure and filament assembly"

Do the authors mean undocking by the N-terminal repeats or repeatedly undocking of these domains?

We meant undocking of the domains, and have corrected the sentence to clarify this.

(5) "Together, these findings provide a structural view of full-length LRRK2 in a closed kinaseconformation and capture a resolved snapshot along its conformational continuum"

Needs a space.

We have made this change, and thank the reviewer for pointing it out.

(6) "Microtubule decoration by LRRK2IT has not been studied in cell types that endogenously express high levels of LRRK2, such as lung epithelial cells and brain-resident immune cells including microglia and macrophages44. Thus, it remains possible that aberrant LRRK2microtubule interactions occur under physiological expression conditions, potentially disrupting homeostatic intracellular transport and being further exacerbated by type I LRRK2 inhibitors, as suggested by in vitro studies23,45."

Many studies have studied the localization of endogenous LRRK2, however were not able to detect filament localization on microtubules. Moreover, to my knowledge, there is also no clear evidence that type 1 inhibitors disrupt microtubule transport in cells expressing endogenous levels of LRRK2.

Therefore, I suggest to rephrase or remove this paragraph.

We agree that the current evidence does not establish that this occurs broadly in cells. However, to our knowledge, cells or tissues with high endogenous LRRK2 expression have not yet been systematically examined in this context. We therefore present sparse decoration of hyperactive LRRK2 on microtubules as a possibility rather than a strong conclusion. We have also previously shown that type I inhibitors disrupt microtubule transport in vitro, but determining whether a similar effect occurs in cells is ongoing work and beyond the scope of the present manuscript.

**Reviewer #3 (Recommendations for the authors):**

(1) P4: The first section of the Results refers to LRRK2 localising to microtubules in the presence of the type-I compounds, and to the cytosol with the type-II inhibitor. Aren't microtubules in the cytosol also?

We meant cytosolic LRRK2, we have revised the text to reflect this. It now reads:

In cells treated with MLi-2, we observed LRRK2^IT^ in extended filaments, puncta, and diffuse in the cytosol (Fig. 1D-E; Ext. Fig 1A-D). In contrast, when cells were treated with GZD-824, LRRK2^IT^ was mostly localized to puncta and distributed throughout the cytosol, with reduced filament formation (Fig. 1F-G; Ext. Fig 1E-H), in agreement with our previous work [23,24,40].

(2) P4: second column, halfway down. I don't understand how the 16 and 8 neighbours are derived from Figure 3J-K. Perhaps indicate this in the figure?

Thank you for bringing this to our attention. We have added an Extended Data Figure 5 to clarify this point. The Extended data figure 5 highlights and annotates the immediate neighboring LRRK2 densities in the MLi-2- and GZD-824-treated lattices, making clear how the 16 and 8 nearest-neighbor values were assigned from the observed lattice organization.

(3) P6: first column, halfway down: perhaps make it explicit that only rigid-body fitting was performed because of the limited resolution?

We have incorporated this useful suggestion. The text now reads:

“We split this model in three parts: the WD40 and C-lobe of the kinase, the N-lobe of the kinase with ROC and COR domains, and the LRR and ANK domains, aligned and fitted each of these three to our map A (Fig. 4D-F). Given the limited resolution of the map A, we fit the model as three rigid bodies without atomic refinement.”

(4) P6: same column near the bottom: what is map C? and how was it calculated? Also, it is not clear to me from Figures 5D-F whether the statement "clearly correspond to the LRR-ANK-ARM domains" is justified by the map. From Figure 5D-F, I see a rather poor fit in a low-resolution map. This needs to be toned down or better illustrated.

We apologize for the oversight. We have updated the text to clarify how the map C was calculated. Now the text reads:

“Additionally, we performed subtomogram analysis in Dynamo on a larger LRRK2^IT^decorated lattice that contained three layers of LRRK2^IT^ density around the microtubule; we refer to this average as map C. Refinement was focused on the central four LRRK2^IT^ subunits to better resolve additional protein densities within this larger lattice. In map C (Fig. 5A; Ext. Fig. 7).”

In addition, we updated the figure 5D-F to better demonstrate the fit of the N-terminal domains into the presented map. We also added an Extended Data Figure 7 to the supplemental materials to further highlight the fit within the map and indicate the areas that correspond to the N-terminal domains of LRRK2. We hope these updates clarify how map C was calculated and better illustrate our interpretation of the additional densities.
